# Generation of twenty four induced pluripotent stem cell lines from twenty four members of the Lothian Birth Cohort 1936

**DOI:** 10.1016/j.scr.2020.101851

**Published:** 2020-07

**Authors:** Jamie Toombs, Lindsay Panther, Loren Ornelas, Chunyan Liu, Emilda Gomez, Raquel Martín-Ibáñez, Simon R. Cox, Stuart J. Ritchie, Sarah E. Harris, Adele Taylor, Paul Redmond, Tom C. Russ, Lee Murphy, James D. Cooper, Karen Burr, Bhuvaneish T. Selvaraj, Cathy Browne, Clive N. Svendsen, Sally A. Cowley, Ian J. Deary, Siddharthan Chandran, Tara L. Spires-Jones, Dhruv Sareen

**Affiliations:** a Centre for Discovery Brain Sciences, UK Dementia Research Institute, The University of Edinburgh, UK; b iPSC Core, The David Janet Polak Foundation Stem Cell Core Laboratory, Cedars-Sinai Medical Center, Los Angeles, CA 90048, USA; c Cedars-Sinai Biomanufacturing Center, West Hollywood, CA 90069, USA; d Lothian Birth Cohorts, Department of Psychology, University of Edinburgh, Edinburgh, UK; e Alzheimer Scotland Dementia Research Centre, University of Edinburgh, Edinburgh, UK; f Edinburgh Clinical Research Facility, University of Edinburgh, Edinburgh, UK; g Dementia Research Institute at the University of Edinburgh, UK; h Board of Governors-Regenerative Medicine Institute, Cedars-Sinai Medical Center, Los Angeles, CA 90048, USA; i Department of Biomedical Sciences, Cedars-Sinai Medical Center, Los Angeles, CA, 90048, USA; j James Martin Stem Cell Facility, Sir William Dunn School of Pathology, University of Oxford, Oxford, UK; k Oxford Parkinson’s Disease Centre, Oxford, UK; l Centre for Clinical Brain Sciences, University of Edinburgh, Edinburgh, Scotland, UK

## Abstract

Cognitive decline is among the most feared aspects of ageing. We have generated induced pluripotent stem cells (iPSCs) from 24 people from the Lothian Birth Cohort 1936, whose cognitive ability was tested in childhood and in older age. Peripheral blood mononuclear cells (PBMCs) were reprogrammed using non-integrating oriP/EBNA1 backbone plasmids expressing six iPSC reprogramming factors (OCT3/4 (POU5F1), SOX2, KLF4, L-Myc, shp53, Lin28, SV40LT). All lines demonstrated STR matched karyotype and pluripotency was validated by multiple methods. These iPSC lines are a valuable resource to study molecular mechanisms underlying individual differences in cognitive ageing and resilience to age-related neurodegenerative diseases.

## 1 Resource Table

**Table:** 

Unique stem cell lines identifier	EDi021-A EDi022-A EDi023-A EDi025-A EDi026-A EDi027-A EDi028-A EDi029-A EDi030-A EDi031-A EDi032-A EDi033-A EDi034-A EDi035-A EDi036-A EDi037-A EDi038-A EDi039-A EDi040-A EDi041-A EDi042-A EDi043-A EDi044-A EDi045-A
Alternative names of stem cell lines	N/A
Institution	Cedars-Sinai Medical Center, Los Angeles, USA
Contact information of distributor	USA distributer: Dhruv Sareen – dhruv.sareen@cshs.org UK distributor: Karen Burr – Karen.burr@ed.ac.uk Clinical data distributor: Paul Redmond – paul.redmond@ed.ac.uk
Type of cell lines	iPSC
Origin	Human
Cell Source	Peripheral Blood Mononuclear Cell
Clonality	Clonal
Method of reprogramming	Non-integrating episomal plasmids
Multiline rationale	24 cell lines from a shared birth year/region cohort
Gene modification	NO
Type of modification	N/A
Associated disease	N/A
Gene/locus	N/A
Method of modification	N/A
Name of transgene or resistance	N/A
Inducible/constitutive system	N/A
Date archived/stock date	EDi021-A: 14/07/2017 EDi022-A: 26/04/2017 EDi023-A: 29/03/2017 EDi025-A: 23/02/2018 EDi026-A: 30/06/2017 EDi027-A: 03/05/2017 EDi028-A: 14/06/2017 EDi029-A: 28/07/2017 EDi030-A: 19/05/2017 EDi031-A: 21/03/2018 EDi032-A: 18/01/2017 EDi033-A: 31/08/2016 EDi034-A: 16/12/2016 EDi035-A: 22/03/2017 EDi036-A: 13/01/2017 EDi037-A: 24/02/2017 EDi038-A: 23/06/2017 EDi039-A: 06/06/2018 EDi040-A: 21/06/2017 EDi041-A: 18/08/2017 EDi042-A: 14/06/2017 EDi043-A: 03/02/2017 EDi044-A: 03/05/2017 EDi045-A: 21/02/2018
Cell line repository/bank	The following lines have been added to the Cedars-Sinai iPSC Core Repository which can be viewed by the public online at https://biomanufacturing.cedars-sinai.org. Direct links to each database record are included below. EDi021-A (Link) EDi022-A (Link) EDi023-A (Link) EDi025-A (Link) EDi026-A (Link) EDi027-A (Link) EDi028-A (Link) EDi029-A (Link) EDi030-A (Link) EDi031-A (Link) EDi032-A (Link) EDi033-A (Link) EDi034-A (Link) EDi035-A (Link) EDi036-A (Link) EDi037-A (Link) EDi038-A (Link) EDi040-A (Link) EDi041-A (Link) EDi042-A (Link) EDi043-A (Link) EDi044-A (Link) EDi045-A (Link)
Ethical approval	NHS Lothian Research Ethics Committee: 10/S1103/10. CSMC Induced Pluripotent Stem Cell (iPSC) Core Facility Repository and Stem Cell program IRB Protocol: Pro00032834

### 1.1 Resource utility

The neurobiology of cognitive ability and its decline during ageing are poorly understood. Human iPSC lines from the Lothian Birth Cohort 1936 comprise individuals with rich life-course cognitive performance data (Taylor et al., 2018, Wardlaw et al., 2011), affording a rare model to investigate molecular mechanisms relevant to differences in brain development, cellular resilience, and vulnerability to pathology.

### 1.2 Resource details

Human peripheral blood mononuclear cells (PBMCs) were obtained from 24 unrelated members of the Lothian Birth Cohort 1936. Demographic parameters are 50% female (n = 12), 100% white Scottish (Table 1). Line donors can be grouped into ‘successful’, ‘typical’, and ‘poor’ cognitive ageing categories (sFig. 1). Exclusion criteria were: self-reported dementia, Parkinson’s disease or stroke, Mini Mental State Examination (MMSE score <24, as well as standardised childhood IQ scores (<65, Moray House Test No. 12 at age 11), and standardised adult IQ scores (<85, average of Moray House Test No. 12 at age 70 and 76).

**Table 1:** Summary of lines.

iPSC line names	Abbreviation in figures	Gender	Age at collection	Ethnicity	Genotype of locus	Disease
EDi021-A		M	78.8	White Scottish	N/A	N/A
EDi022-A		M	79.22	White Scottish	N/A	N/A
EDi023-A		F	79.1	White Scottish	N/A	N/A
EDi025-A		M	78	White Scottish	N/A	N/A
EDi026-A		M	79.45	White Scottish	N/A	N/A
EDi027-A		F	79.65	White Scottish	N/A	N/A
EDi028-A		M	79.1	White Scottish	N/A	N/A
EDi029-A		M	80.13	White Scottish	N/A	N/A
EDi030-A		F	78.98	White Scottish	N/A	N/A
EDi031-A		F	78	White Scottish	N/A	N/A
EDi032-A		F	79.29	White Scottish	N/A	N/A
EDi033-A		F	78.67	White Scottish	N/A	N/A
EDi034-A		F	78.68	White Scottish	N/A	N/A
EDi035-A		F	78.79	White Scottish	N/A	N/A
EDi036-A		F	79.22	White Scottish	N/A	N/A
EDi037-A		M	79.1	White Scottish	N/A	N/A
EDi038-A		M	79.19	White Scottish	N/A	N/A
EDi039-A		M	78	White Scottish	N/A	N/A
EDi040-A		M	79.67	White Scottish	N/A	N/A
EDi041-A		F	80.13	White Scottish	N/A	N/A
EDi042-A		F	79.42	White Scottish	N/A	N/A
EDi043-A		M	80.26	White Scottish	N/A	N/A
EDi044-A		F	79.85	White Scottish	N/A	N/A
EDi045-A		M	80.32	White Scottish	N/A	N/A

PBMCs were reprogrammed to generate induced pluripotent stem cells (iPSCs) using episomal plasmids encoding human OCT3/4 (POU5F1), SOX2, KLF4, L-Myc, shp53, Lin28, SV40LT. All lines were reprogrammed and stored within 22 months of each other. EBNA-related gene analysis demonstrated that iPSCs were EBNA transgene-free (and therefore exogenous reprogramming factors were no longer present) by passage 17–21 (depending on line). Qualitative tests for parental cell type by TCR-αβ and TCR-γδ T-cell clonality assay revealed that 83% (n = 20) of lines were non-T cell-derived, 17% (n = 4) were T-cell derived. T-cell derived lines are: EDi021-A, EDi025-A, EDi026-A, and EDi035-A. All lines have been confirmed mycoplasma negative (sFig. 27).

**Fig. 1:**
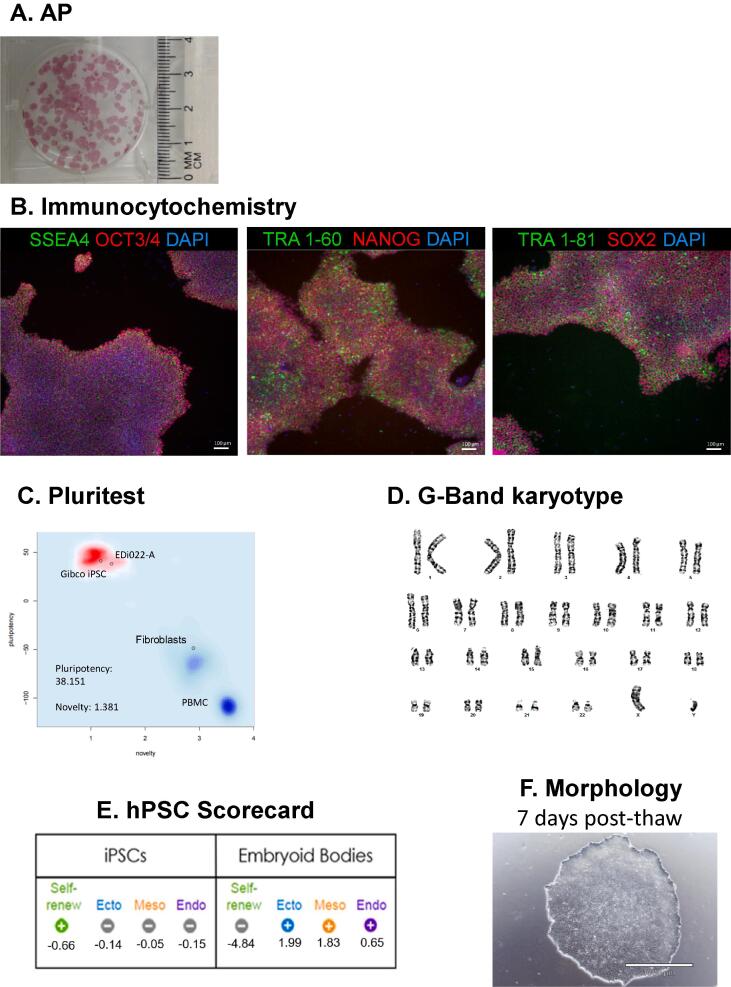
Characterization for iPSC line EDi021-A.

All lines demonstrated stem cell-like morphology (Fig. 1F, sFig2–24F) and expressed six pluripotency markers (OCT3/4, NANOG, SOX2, TRA-1-60, TRA-1-81, SSEA4) evaluated by immunocytochemistry (Fig. 1B, sFig. 2–24B). Additionally, all lines demonstrated positive alkaline phosphatase AP staining (Fig. 1A, sFig. 2–24A) and self-renewal in undifferentiated iPSCs as assessed by PluriTest (Fig. 1C, sFig. 2–24C) and TaqMan®hPSC Scorecard™ Panel (Fig. 1D, sFig. 2–24E). However, whilst EDi035-A had a positive PluriTest and Scorecard^TM^ pluripotency result, the PluriTest novelty score was borderline (1.688) (sFig. 14C, E). Furthermore, EDi027-A also had a borderline positive ectoderm score as assessed by Scorecard™ (sFig. 6E). At 14 days of embryoid body differentiation, all lines demonstrated tri-lineage potential except EDi022-A (negative endoderm, borderline mesoderm score, sFig. 2E), EDi035-A (negative mesoderm, borderline endoderm score, sFig. 14E), and EDi042-A (negative endoderm score, sFig. 21E), as assessed by Scorecard™.

All lines showed a normal karyotype (Fig. 1D, sFig. 2–24D) between passages 6–22, with one exception. All five clones of EDi-038-A (a male) karyotyped as monosomy (45,X) (sFig. 18D), and thus very likely stems from the source PBMCs. Mosaicism is a relatively common and probably harmless finding in blood cultures from normal females and, though rarer, also in males (Bukvic et al., 2001). No differences were detected between the original PBMC samples and the corresponding iPSC lines.

All lines were confirmed to be of human origin and iPSCs matched the profile of parent PBMCs by Short Tandem Repeat (STR) analysis. Parent line data was not available for EDi026-A and EDi028-A. Genetic profiles for these lines were compared to the cell line genetic profiles available in the DSMZ STR database and did not match any other reported profiles in the DSMZ database. These profiles were found to be unique and did not match to any previously submitted profiles from the iPSC Core. The genetic profiles established here can be used for future comparisons for these cell lines. Whole genome sequence data for all 24 lines has been deposited at the European Genome-phenome Archive (EGA), which is hosted by the EBI and the CRG, under accession number EGAS00001003819.

An overview of iPSC line characterisation can be found in Table 2. Fig. 1 presents example characterisation data from EDi021-A. Data for all other lines can be found in Supplementary Figs. 2–27.

**Table 2:** Characterization and validation.

Classification	Test	Result	Data
Morphology	Photography of phase contrast.	Normal. Colonies of small rounded cells with large nuclei.	Fig. 1F; Supplementary Figures 2-24F.
Phenotype	Qualitative analysis: Immunofluorescence, Alkaline Phosphatase Staining	OCT3/4+, NANOG+, SOX2+, TRA-1–60+, TRA-1–81+, SSEA4+, Alkaline Phosphatase+	Fig. 1A, B; Supplementary Figures 2-24A,B.
	Quantitative analysis: Pluritest	Pluripotency score ≥ 20 and novelty score ≤ 1.6	Fig. 1C; Supplementary Figs. 2–24C
Genotype	Karyotype (G-banding)	Normal XX and XY corresponding to gender (Table 1). Resolution 400 bands.	Fig. 1D; Supplementary Figs. 2–24D
Identity	STR analysis	9 loci tested. 100% match for lines where original PBMCs were available (22/24 lines).	Available with the authors.
		N/A	N/A
Mutation analysis (IF APPLICABLE)	Sequencing.	N/A	N/A
	Southern Blot OR WGS.	N/A	N/A
Microbiology and virology	Mycoplasma.	Negative.	sFig. 27
Differentiation potential	TaqMan® hPSC Scorecard™ Assay.	Endoderm, mesoderm, ectoderm negative at day 0, positive at day 14.	Fig. 1E; Supplementary Figs. 2–24E
Donor screening (OPTIONAL)	HIV 1 + 2 Hepatitis B, Hepatitis C.	N/A	N/A
Genotype additional info (OPTIONAL)	Blood group genotyping.	N/A	N/A
	HLA tissue typing.	N/A	N/A

## 2 Materials and methods

### 2.1 PBMC isolation

Blood samples were collected with NHS Lothian Research Ethics Committee Approval (10/S1103/10). Blood samples were collected in Sodium Citrate BD Vacutainer CPT tubes (BD, Cat. 362761) (three tubes per participant). For samples EDi021-A, EDi025-A, EDi028-A, EDi030-A, EDi031-A, EDi032-A, EDi033-A, EDi034-A, and EDi035-A PBMC isolation was performed by Roslin Cells. For all other lines, PBMC isolation was performed by the Edinburgh Clinical Research Facility (ECRF).

### 2.2 Generation of human iPSCs

Generation of human iPSCs lines from PBMCs was performed using nucleofection of episomal plasmids containing POU5F1, SOX2, KLF4, LIN28, L-MYC, TP53shRNA, and SV40LT.

Briefly, ~5 × 10^6^ cells per nucleofection of PBMCs were nucleofected with the Amaxa Human T-cell Nucleofector® Kit (Lonza, Cat. VVPA-1002) and a 5p plasmid mixture using program V-024 on a Amaxa Nucleofector 2D Device (Lonza, Cat. AAB-1001). Each transfection contained the following seven factors: OCT4, SOX2, KLF4, LMYC, LIN28, SV40LT and p53 shRNA. These were delivered on the following plasmids from Addgene, together with an EBNA1 plasmid for episomal plasmid maintenance: pEP4 E02S ET2K (Cat. 20927), pCXLE-hOCT3/4-shp53-F (Cat. 27077), pCXLEhUL (Cat. 27080), pCXLE-hSK (Cat. 27078), and pCXWB-EBNA1 (Cat. 37624). Each transfection used 0.5 µg of plasmid pCXWB-EBNA1 and 0.83 µg of each of the remaining four plasmids. After nucleofection, cells were immediately plated in either αβ T-cell medium (X-vivo10 [Lonza, Cat. 04-380Q] supplemented with 30 U/ml IL-2 [ThermoFisher Scientific, Cat. PHC0026] and 5 µl/well Dynabeads Human T-activator CD3/CD28 [Life Technologies, Cat. 11161D]) or non T-cell medium (αMEM [Life Technologies, Cat. 12561056] supplemented with 10% Heat Inactivated-FBS [Life Technologies, Cat. 10437028], 10 ng/ml IL-3 [StemCell Technologies, Cat. 78040.1], 10 ng/ml IL-6 [StemCell Technologies, Cat. 78050.1], 10 ng/ml G-CSF [StemCell Technologies, Cat. 78012.1] and 10 ng/ml GM-CSF [StemCell Technologies, Cat. 78015.1]) onto mitomycin treated mouse embryonic fibroblasts (MEF) and placed in a 37 °C incubator with 20% O2 and 5% CO2.

Two days after nucleofection, 2 mL/well of Primate ESC medium (ReproCell, Cat. RCHEMD001) containing 5 ng/ml bFGF (for MEF condition) was added to the wells without aspirating the previous medium. Beginning on day four, the medium was gently aspirated from each well and 2 mL of the appropriate fresh reprogramming media was added to each well. Medium was replaced every other day. At approximately day 18 post nucleofection, individual colonies were observed in all wells of each condition. Individual PBMC-iPSC colonies with ES/iPSC-like morphology appeared between day 25–32 and those with best morphology were mechanically isolated, transferred onto 12-well plates with fresh Matrigel™ Matrix (Corning/BD Biosciences, Cat. 354230), and maintained in mTeSR®1 medium (StemCell Technologies, Cat. 85850). The iPSC clones were further expanded and scaled up for further analysis. All cultures were maintained at 37 °C, 20% O_2_, and 5% CO_2_ throughout the reprogramming process.

### 2.3 iPSC maintenance and storage

Human iPSCs were cultured in mTeSR®1 medium (StemCell Technologies, Cat. 85850) on growth factor-reduced Matrigel™ Matrix (Corning, Cat. 354230) -coated plates at 37 °C in a 20% O_2_, 5% CO_2_ incubator. Briefly, 70–90% confluent human iPSC colonies were passaged every 7 days chemically (Versene, Life Technologies, Cat. 15040-066 or ReLeSR, StemCell Technologies, Cat. 05872) or mechanically by StemPro® EZPassage™ Disposable Stem Cell Passaging Tool (Life Technologies, Cat. 23181–010) and re-plated at a 1:6 or 1:9 ratio depending on the cell line. The iPSCs were passaged every 5–7 days. The iPSCs were expanded for 6–22 passages during which period various characterization assays were performed. The iPSCs were cryopreserved using CryoStor CS10 (StemCell Technologies, Cat. 07930) and an isopropanol freezing vessel at −80 °C overnight. The cryopreserved vials were subsequently stored in liquid nitrogen tanks for long-term storage. Working Cell Banks (WCB) of iPSCs were cryopreserved at passage 9–14 and then Distribution Cells Banks (DCB) were created between passages 18–22.

### 2.4 Mycoplasma testing

The absence of mycoplasma contamination in the iPSC lines were confirmed monthly using the MycoAlert Detection Kit, a selective biochemical test (LONZA, Cat. LT07-1188).

### 2.5 EBNA-related gene analysis

250 ng of genomic DNA was extracted using the KingFisherTM DUO Prime purification system (Thermo Fisher Scientific) and the MagMAXTM DNA Multi-Sample Ultra 2.0 Kit (Applied Biosystems, A36570). An embryonic stem cell line (H9) was included alongside LBC lines a negative control. DNA Amplification was conducted using TaKaRa Ex Taq® DNA Polymerase (TaKaRa Bio, RR001) and a Bio Rad 1000 Touch Thermal Cycler. Primers that recognize EBNA1 along with housekeeping gene Glyceraldehyde 3-phosphate dehydrogenase (GAPDH), which was used as a housekeeping gene, were included in this study (Table 2). PCR was run for 35 cycles at 95 °C for 30 s, 60 °C for 30 s, and 72 °C for 30 s.

### 2.6 TCRB and TCRG T-Cell Clonality assay

TCRB and TCRG T-Cell Clonality testing was conducted using Gene Rearrangement and Translocation assays from Invivoscribe Technologies, Inc. Genomic DNA was harvested from all iPSC lines using the MagMAX™ DNA Multi-Sample Ultra 2.0 Kit (Cat. A36570) from Applied Biosystems and it was re-suspended to a final concentration of 100–400 μg per ml in dilution buffer. Three Clonal Control DNA and one Polyclonal Control DNA provided with the kit were used. PCR was carried out as per the manufacturer’s protocol. PCR products were analysed using 6% TBE gel electrophoresis with gel red staining.

### 2.7 Karyotyping

Human PBMC-iPSCs were incubated in Colcemid (100 ng/mL; Life Technologies) for 30 min at 37 °C and then dissociated using TrypLE for 5 min. They were then washed in phosphate buffered saline (PBS) and incubated at 37 °C in 5 mL hypotonic solution (1 g KCl, 1 g Na Citrate in 400 mL water) for 30 min. The cells were centrifuged for 2.5 min at 1500RPM and re-suspended in fixative (methanol: acetic acid, 3:1) at room temperature for 5 min. This was repeated twice, and finally cells were re-suspended in 500 μl of fixative solution and submitted to the Cedars-Sinai Clinical Cytogenetics Core for G-band karyotyping. Karyotyping of each iPSC line was conducted at early and late passage, between passages 6–22. Approximately 20 metaphase spreads were counted per line.

### 2.8 Immunocytochemistry

iPSCs were plated on Matrigel™ (Corning, Cat. 354230) -coated glass coverslips or optical-bottom 96-well plates (ThermoFisher Scientific, Cat. 165305) and subsequently fixed in 4% paraformaldehyde (10 min, room temperature (RT)). The blocking buffer used was 5% goat serum (Millipore, Cat. S26-100ML) and 5% donkey serum (Millipore, S30-100ML) with 0.15% Triton X-100 in PBS, except for SSEA4 and OCT4 staining, for which 5% goat serum with 0.15% Triton X-100 in PBS was used as the block. All cells were blocked for one hour at RT, then incubated with primary antibodies (Table 3) for either 3 h at RT or overnight at 4 °C. Cells were then rinsed and incubated in species-specific AF488 or AF594-conjugated secondary antibodies (1:500, diluted in the same block as the primary antibodies) for one hour at RT, followed by DAPI (0.5–1 μg/ml; Sigma) to counterstain nuclei (10 min, RT). Cells were imaged using Nikon/Leica microscopes or Image Express. The iPSCs exhibited an embryonic stem cell like morphology, and expressed a range of pluripotency markers (OCT3/4, NANOG, SOX2, TRA-1–60, TRA-1–81, SSEA4) (Fig. 1B, Supplementary Figs. 2–24B).

**Table 3:** Reagents details.

Antibodies used for immunocytochemistry/flow-cytometry			
	Antibody	Dilution	Company Cat # and RRID
Pluripotency Markers	SSEA4 (mlgG3) TRA-1–60 (mlgM,_K_) TRA-1–81 (mlgM,_K_) OCT4 (Rabbit, IgG) NANOG (Rabbit, IgG) SOX2 (Rabbit, IgG)	1:250 1:250 1:250 1:250 1:250 1:250	Stemgent (cat. 09-0006, RRID: AB_1512169) Stemgent (cat. 09-0010, RRID: AB_1512170) Stemgent (cat. 09-0011, RRID: AB_1512171) Stemgent (cat. 09-0023, RRID: AB_2167689) Stemgent (cat. 09-0020, RRID: AB_2298294) Stemgent (cat. 09-0024, RRID: AB_2195775)
N/A	N/A	N/A	N/A
Secondary antibodies	Donkey anti-Mouse IgG AF488 Donkey anti-Rabbit IgG AF594 Goat anti-Mouse IgG, IgM, IgA AF488	1:500	Life Technologies (cat. A-21202) Life Technologies (cat. A-21207) Life Technologies (cat. A-10667)

Primers			
	Target	Forward/Reverse primer (5′–3′)	
Episomal Plasmids (qPCR)	Epstein-Barr virus nuclear antigen (EBNA)	GGTCCCGAGAATCCCCATCC/TTCATGGTCGCTGTCAGACAG	
N/A	N/A	N/A	
House-Keeping Genes (qPCR)	Glyceraldehyde 3-phosphate dehydrogenase (GAPDH)	GTGGACCTGACCTGCCGTCT/ GGAGGAGTGGGTGTCGCTGT	
N/A	N/A	N/A	
N/A	N/A	N/A	

### 2.9 Alkaline phosphatase staining

Alkaline phosphatase staining was performed using the Alkaline Phosphatase Staining Kit II (Stemgent, Cat. 00–0055) according to the manufacturer's instructions.

### 2.10 PluriTest

PluriTest was used to assess the pluripotency of undifferentiated iPSCs (Fig. 1C, Supplementary Figs. 2–24C). Cell pellets were sent to Life Technologies Corporation for the PluriTest Service. Total RNA was isolated using the PureLinkTM RNA Mini Kit (Thermo Fisher Scientific) and quantified using NanoDropTM. 100 ng total RNA was used to prepare the GeneChip® for the PluriTest™. In this assay, 36,000 transcripts and variants against a >450 sample reference set are assessed for gene expression analysis. A non-iPSC sample was used in this experiment to serve as a control for non-pluripotency. The transcriptome of all samples were analysed and processed in the PluriTest™ algorithm to generate a pluripotency and novelty score. These two scores determine the pluripotency signature of the cell line which is represented in the pluripotency plot. The threshold for pluripotency was >20, and the threshold for novelty was <1.6.

### 2.11 hPSC Scorecard data analysis

Applied Biosystems TaqMan®hPSC Scorecard™ Panel (Thermo Fisher Scientific) was used as an additional technique to assess pluripotency and tri-lineage differentiation potential of iPSC lines using real-time qPCR assays (Fig. 1E, Supplementary Figs. 2–24E). Total RNA from undifferentiated and EB differentiated iPSC lines was isolated using MagMAX™ mirVana™ Total RNA Isolation Kit (A27828), and 1 μg of RNA was used to make cDNA using the High Capacity cDNA Reverse Transcription Kit (4368813), both from Applied Biosystems. TaqMan qRT-PCR was carried out using the hPSC Scorecard 384w Fast plate (Life technologies, A15870) and QuantStudio 12 k Flex, following manufacturer protocol. We analysed the gene expression data from the TaqMan®hPSC Scorecard™ Panel using the web-based hPSC Scorecard™ Analysis Software (Thermo Fisher Scientific).

### 2.12 Embryoid body (EB) formation

IPSC lines were allowed to differentiate by EB formation. Briefly, iPSCs were lifted from 3 wells of a 6 well plate using a cell scraper and seeded in a T25 flask treated with poly-HEMA to prevent cell attachment in EB media containing: IMDM basal media (Cat. 12440061), 17% KnockOut Serum Replacement (KOSR; Cat. 10828028), 1% non-essential amino acids (Cat. 11140050), 1% Antibiotic-Antimycotic (Cat. 15240062) and 110 µM β-Mercaptoethanol (Cat. 21985023), all from Thermo Fisher. EBs were allowed to form by self-aggregation, grow and differentiate for 14 days in EB culture media replacing it twice a week. Differentiation to endoderm, mesoderm and ectoderm was assessed by TaqMan® hPSC Scorecard™ Assay (Fig. 1E, Supplementary Figs. 2–24E).

## 3 STR analysis

Short Tandem Repeat (STR) Analysis is conducted to confirm iPSC genetic identity. For that, a frozen vial of the parent PBMCs and a frozen vial of the reprogramed iPSC line at late passage (18–21, depending on the cell line) are sent to IDEXX BioResearch. STR profile and interspecies contamination testing is analysed. iPSC line human authentication was conducted at IDEXX BioResearch by Cell Check™. Profiling included using a nine marker STR profile (AMEL, CSF1PO, D13S317, D16S539, D5S818, D7S820, TH01, TPOX and vWA) and interspecies contamination check for human, mouse, rat, African green monkey and Chinese hamster cells. Comparative analysis was conducted between parent PBMCs and reprogrammed iPSC lines.

## Declaration of Competing Interest

The authors declare the following financial interests/personal relationships which may be considered as potential competing interests: US patent US 10,221,395 B2 has been granted describing some of the methods to reprogram to iPSCs. Apart from this issued patent filing the authors have declared that no other competing financial interests exist.

## References

[b0005] Bukvic N., Gentile M., Susca F., Fanelli M., Serio G., Buonadonna L., Capurso A., Guanti G. (2001). Sex chromosome loss, micronuclei, sister chromatid exchange and aging: a study including 16 centenarians. Mutat. Res. Toxicol. Environ. Mutagen..

[b0010] Taylor A.M., Pattie A., Deary I.J. (2018). Cohort profile update: the lothian birth cohorts of 1921 and 1936. Int. J. Epidemiol..

[b0015] Wardlaw J.M., Bastin M.E., Valdés Hernández M.C., Maniega S.M., Royle N.A., Morris Z., Clayden J.D., Sandeman E.M., Eadie E., Murray C., Starr J.M., Deary I.J. (2011). Brain aging, cognition in youth and old age and vascular disease in the lothian birth cohort 1936: rationale, design and methodology of the imaging protocol. Int. J. Stroke.

