# Supplementary material for: Generation of twenty four induced pluripotent stem cell lines from twenty four members of the Lothian Birth Cohort 1936
Source: Stem Cell Res. 2020 Jul;46:101851. doi: 10.1016/j.scr.2020.101851 (PMC7347008; doi:10.1016/j.scr.2020.101851)
Supplement: Supplementary data 1 [file mmc1.pdf]

Selection of iPSC line donors based on cognitive ageing

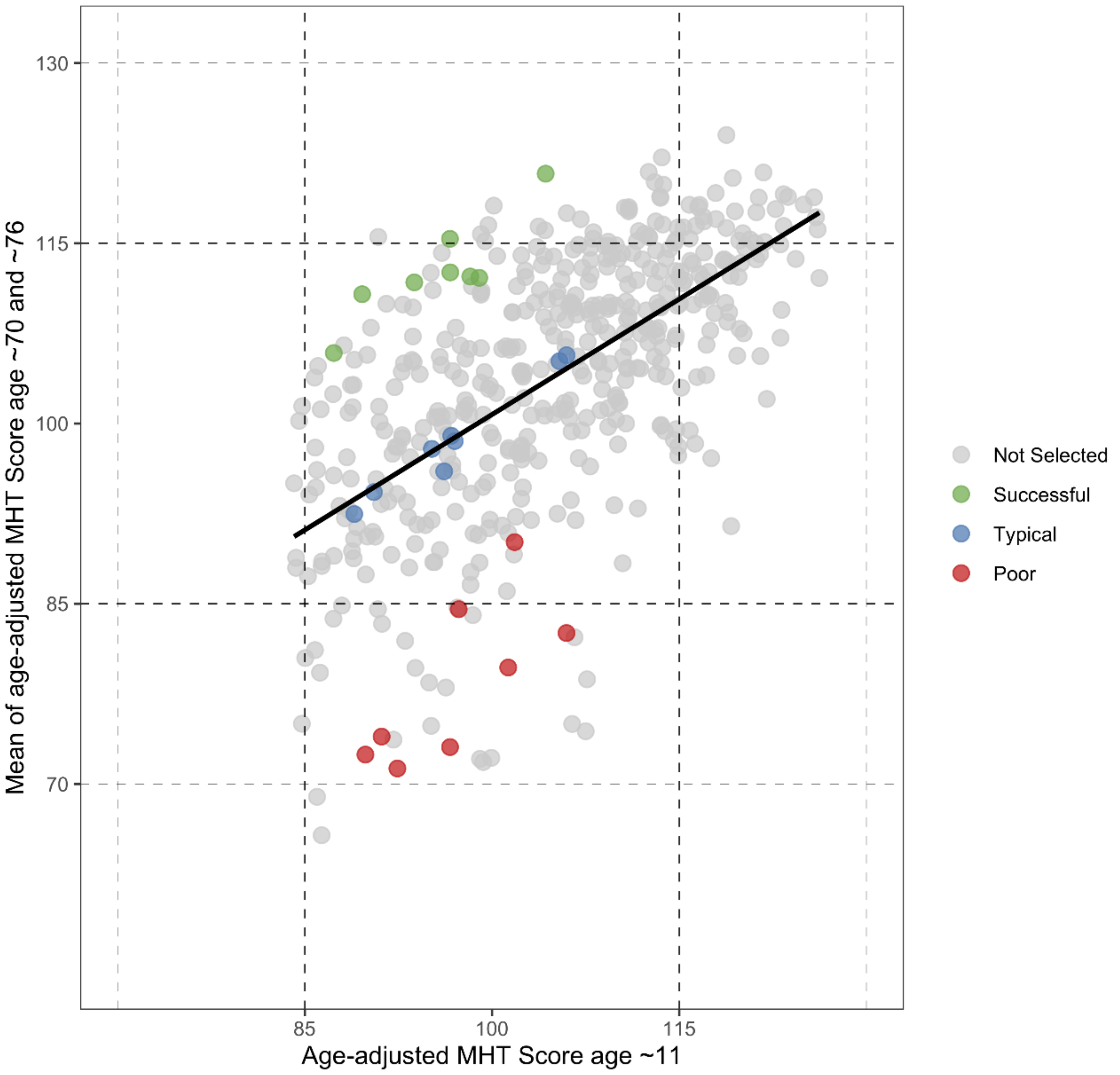

sFig1: Selection of iPSC candidates based on cognitive ageing profiles in the Lothian Birth Cohort 1936. Dashed lines are added to show ±1SD from the mean for the age 11 Moray House Test (MHT) score and ±1 and 2SDs from the mean for later-life MHT score.

sFig.2: Characterization for iPSC line EDi022-A

A. AP

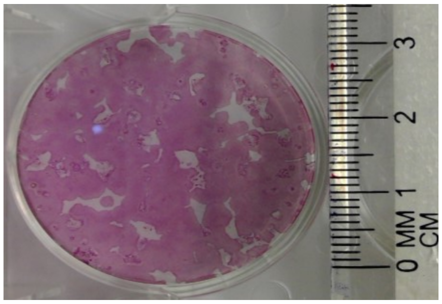

B. Immunocytochemistry

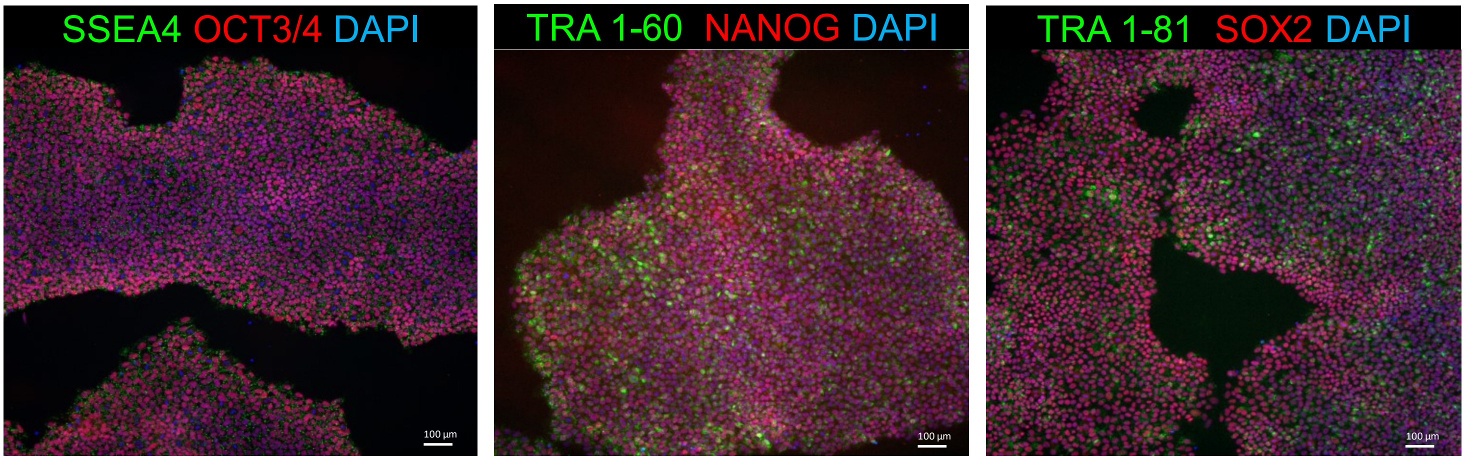

C. PluriTest

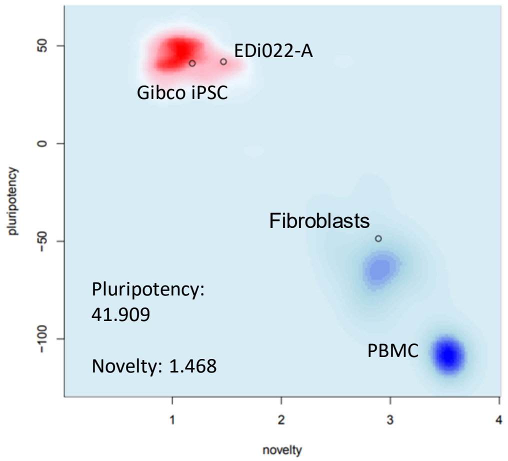

D. G-Band karyotype

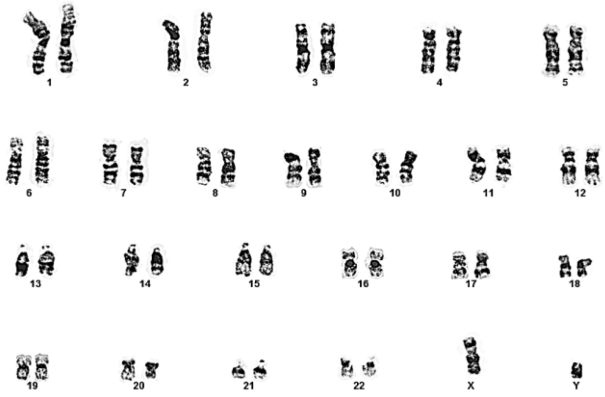

E. hPSC Scorecard

iPSCs				Embryoid bodies			
Self-renew	Ecto	Meso	Endo	Self-renew	Ecto	Meso	Endo
+	−	−	−	−	+	o	−
0.29	-0.10	0.82	-0.53	-3.08	1.41	0.78	-0.32

F. Morphology

3 days post-thaw

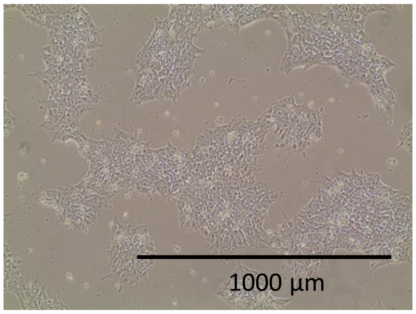

sFig.3: Characterization for iPSC line EDi023-A

A. AP

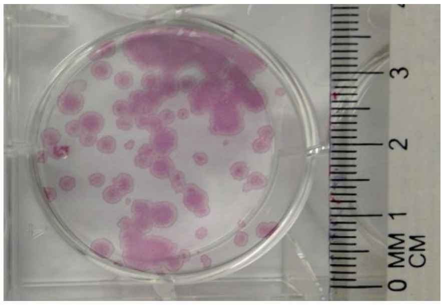

B. Immunocytochemistry

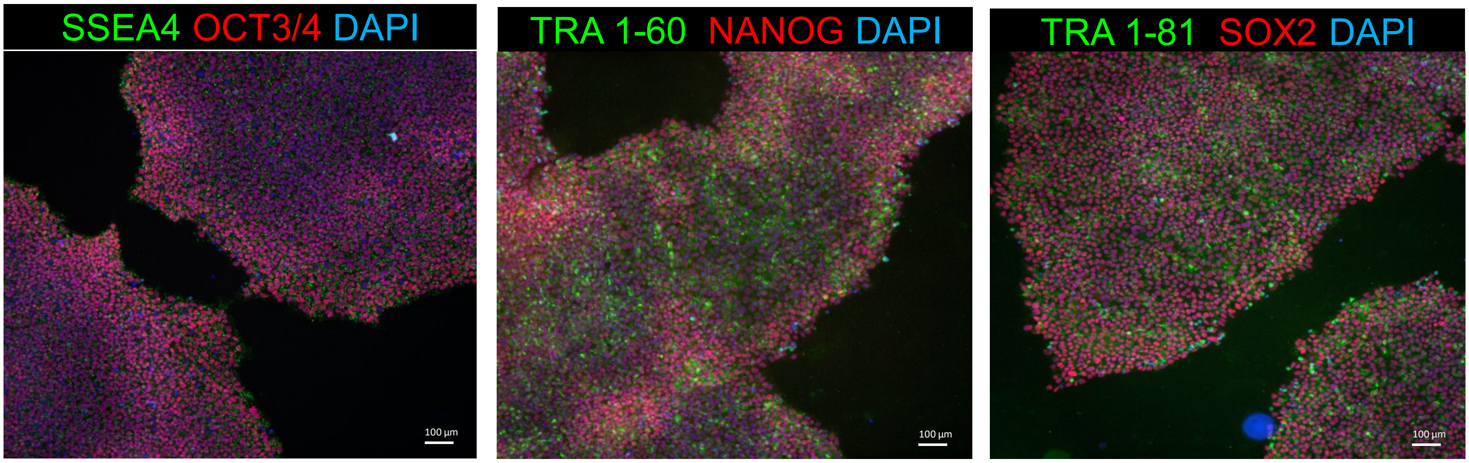

C. PluriTest

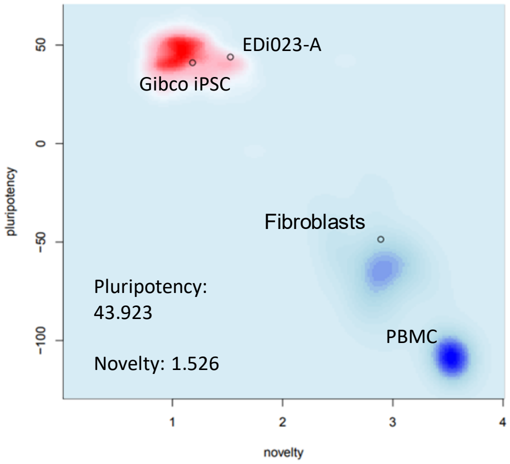

D. G-Band karyotype

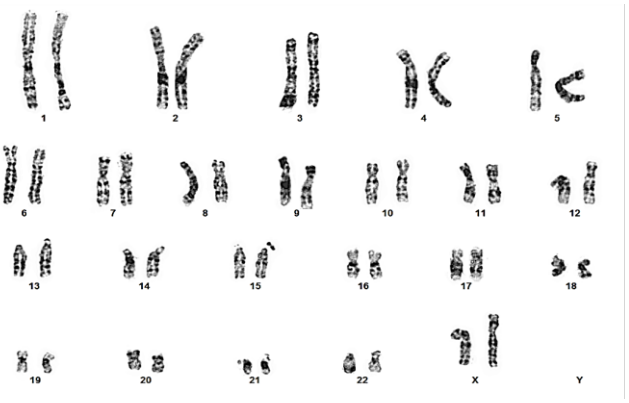

E. hPSC Scorecard

iPSCs				Embryoid bodies			
Self-renew	Ecto	Meso	Endo	Self-renew	Ecto	Meso	Endo
+	−	−	−	−	+	+	+
0.28	-0.30	0.34	-0.67	-3.49	2.38	3.38	1.12

F. Morphology

3 days post-thaw

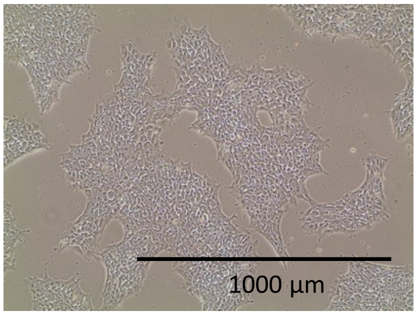

sFig.4: Characterization for iPSC line EDi025-A

A. AP

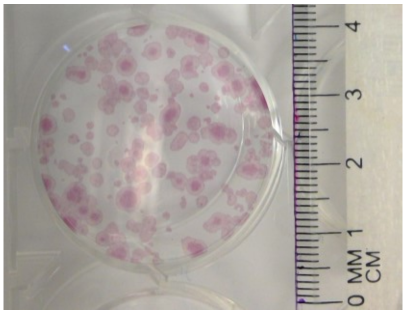

B. Immunocytochemistry

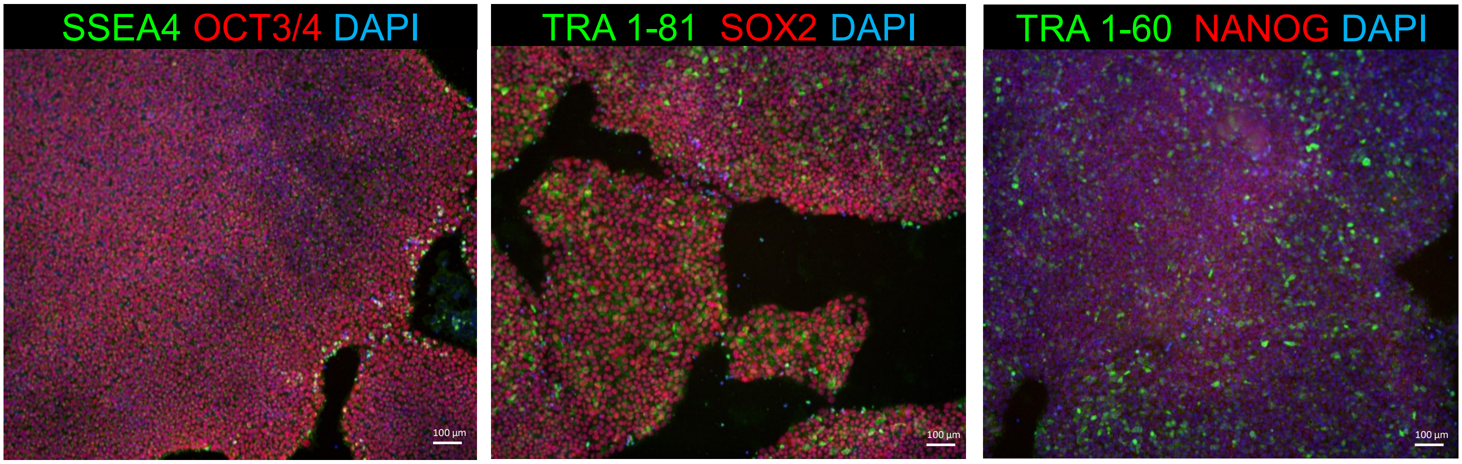

C. PluriTest

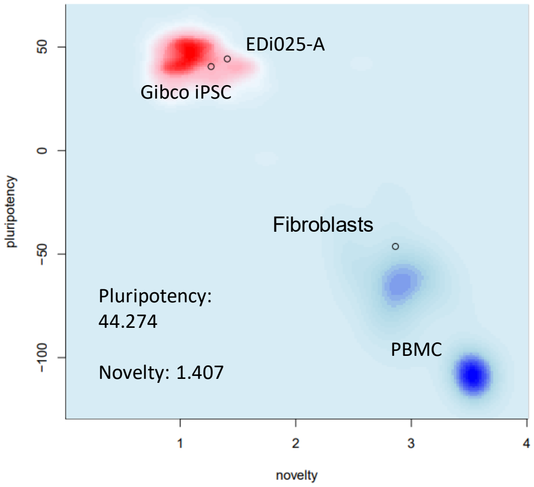

D. G-Band karyotype

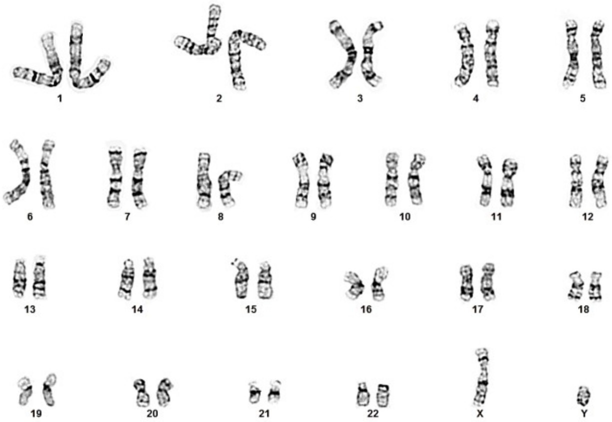

E. hPSC Scorecard

iPSCs				Embryoid Bodies			
Self-renew	Ecto	Meso	Endo	Self-renew	Ecto	Meso	Endo
+	−	−	−	−	+	+	+
-0.01	-0.33	-0.39	-1.15	-7.29	1.49	2.19	0.57

F. Morphology

2 days post-thaw

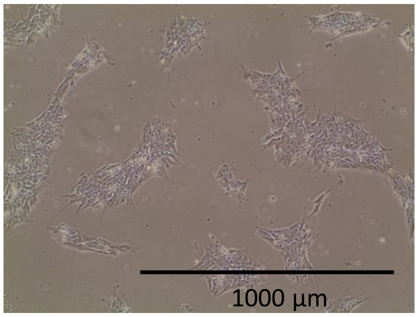

sFig.5: Characterization for iPSC line EDi026-A

A. AP

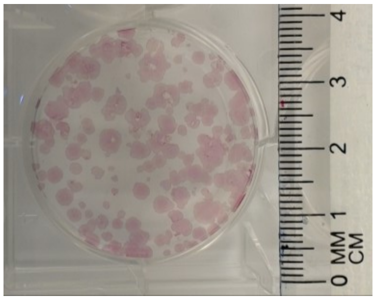

B. Immunocytochemistry

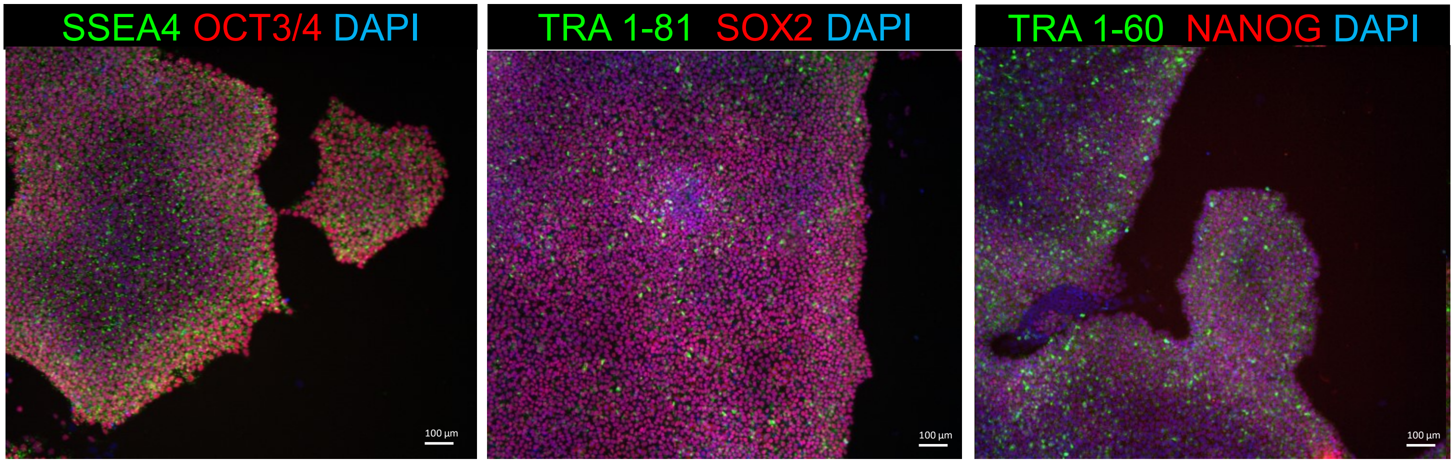

C. Pluritest

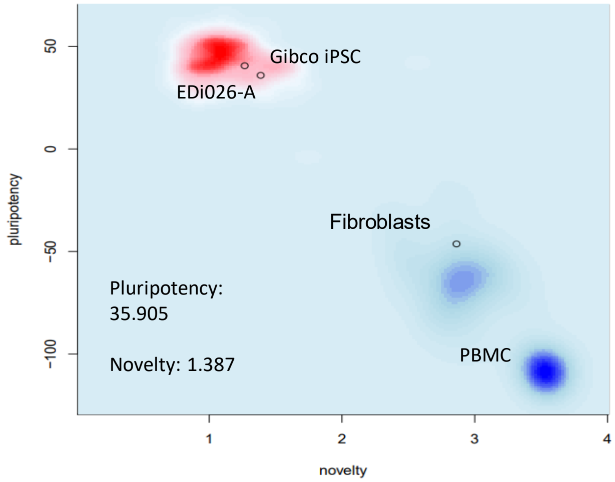

D. G-Band karyotype

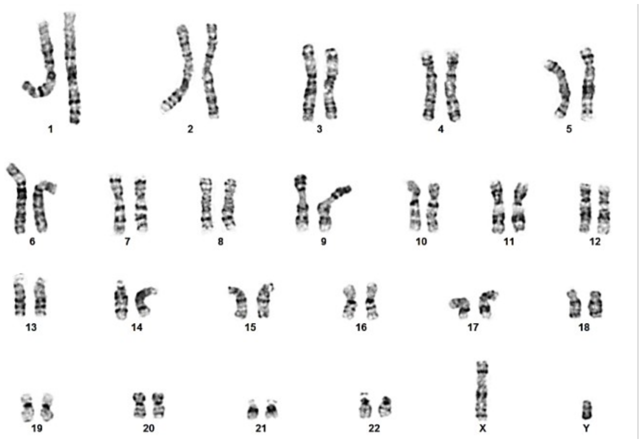

E. hPSC Scorecard

iPSCs				Embryoid Bodies			
Self-renew	Ecto	Meso	Endo	Self-renew	Ecto	Meso	Endo
+	−	−	−	−	+	+	+
-0.41	0.50	0.42	-0.83	-6.72	1.74	1.19	0.69

F. Morphology

2 days post-thaw

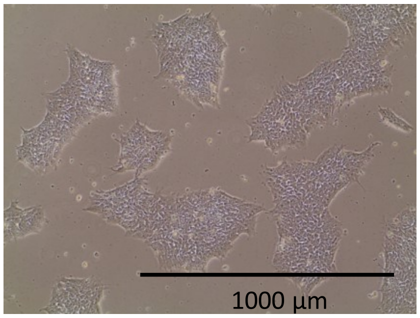

sFig.6: Characterization for iPSC line EDi027-A

A. AP

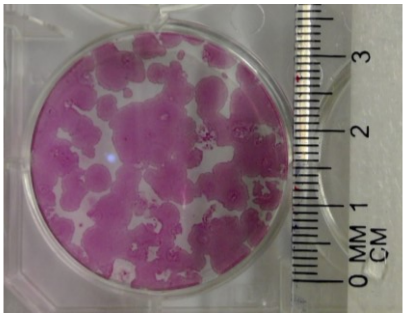

B. Immunocytochemistry

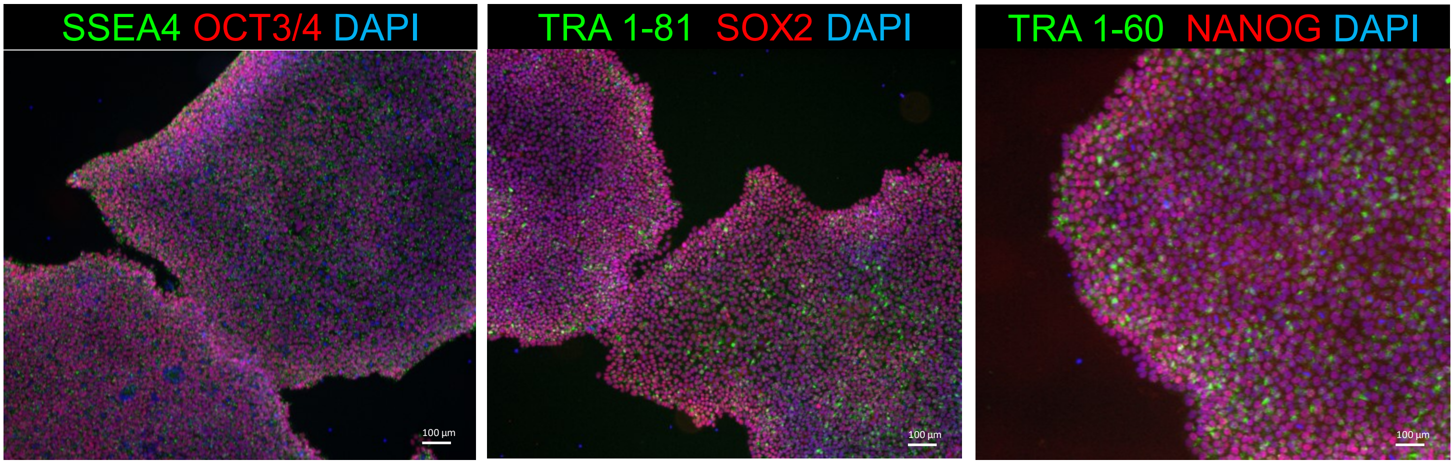

C. Pluritest

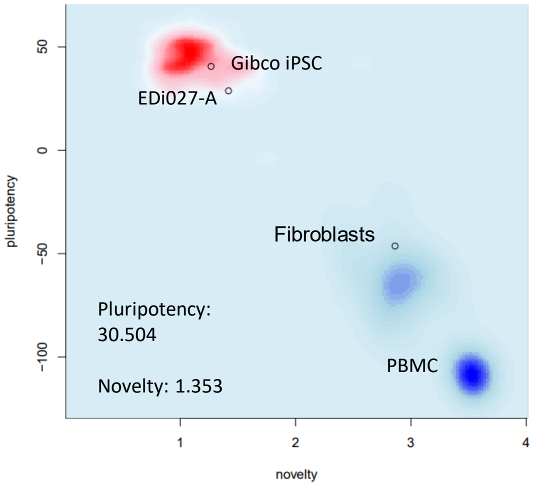

D. G-Band karyotype

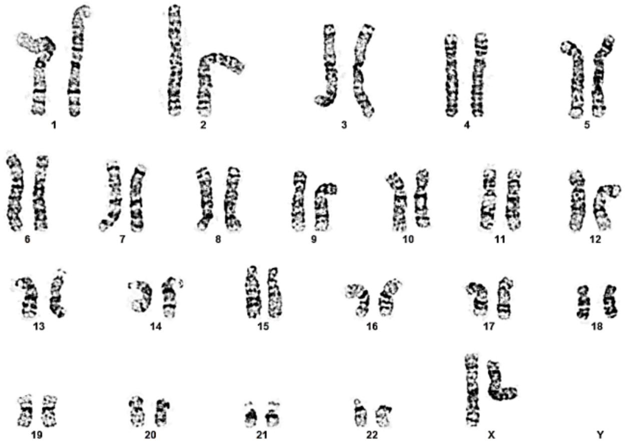

E. hPSC Scorecard

iPSCs				Embryoid Bodies			
Self-renew	Ecto	Meso	Endo	Self-renew	Ecto	Meso	Endo
+	o	−	−	−	+	+	+
0.18	0.61	0.10	-0.75	-6.07	-1.72	-3.29	-1.78

F. Morphology

11 days post-thaw

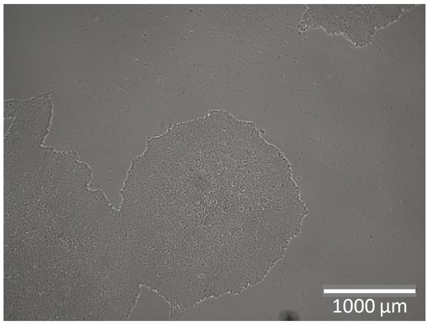

sFig.7: Characterization for iPSC line EDi028-A

A. AP

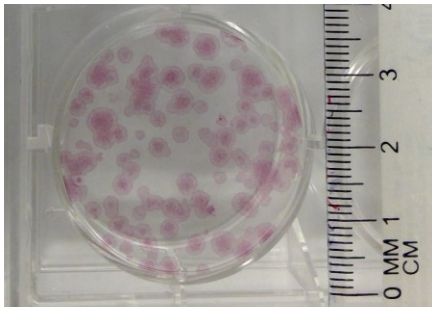

B. Immunocytochemistry

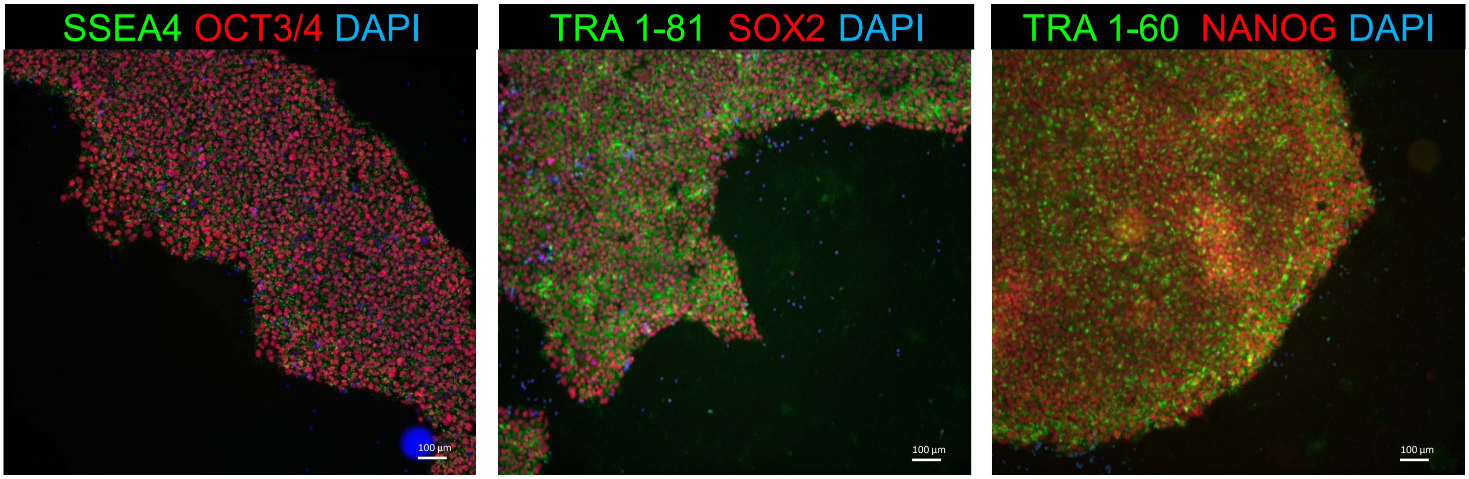

C. Pluritest

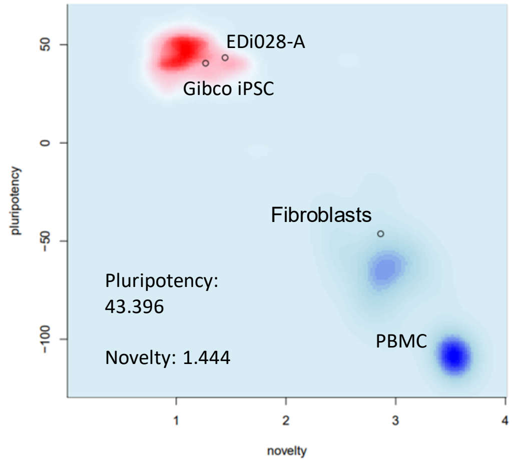

D. G -Band karyotype

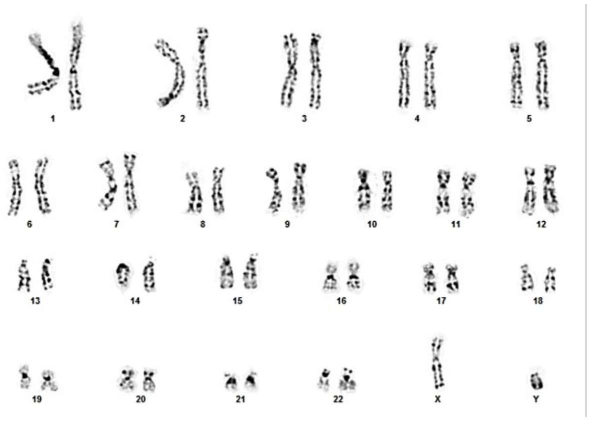

E. hPSC Scorecard

iPSCs				Embryoid Bodies			
Self-renew	Ecto	Meso	Endo	Self-renew	Ecto	Meso	Endo
+	−	−	−	−	+	+	+
-0.17	-0.17	0.69	-1.00	-6.84	2.14	2.10	0.69

F. Morphology

2 days post-thaw

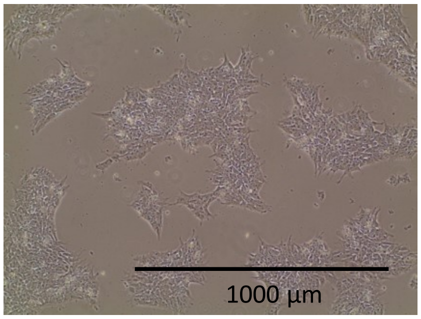

sFig.8: Characterization for iPSC line EDi029-A

A. AP

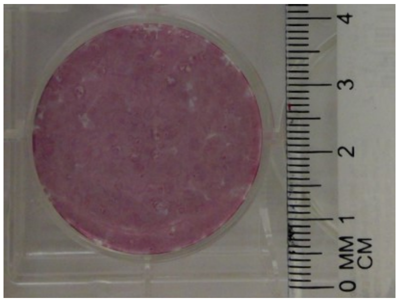

B. Immunocytochemistry

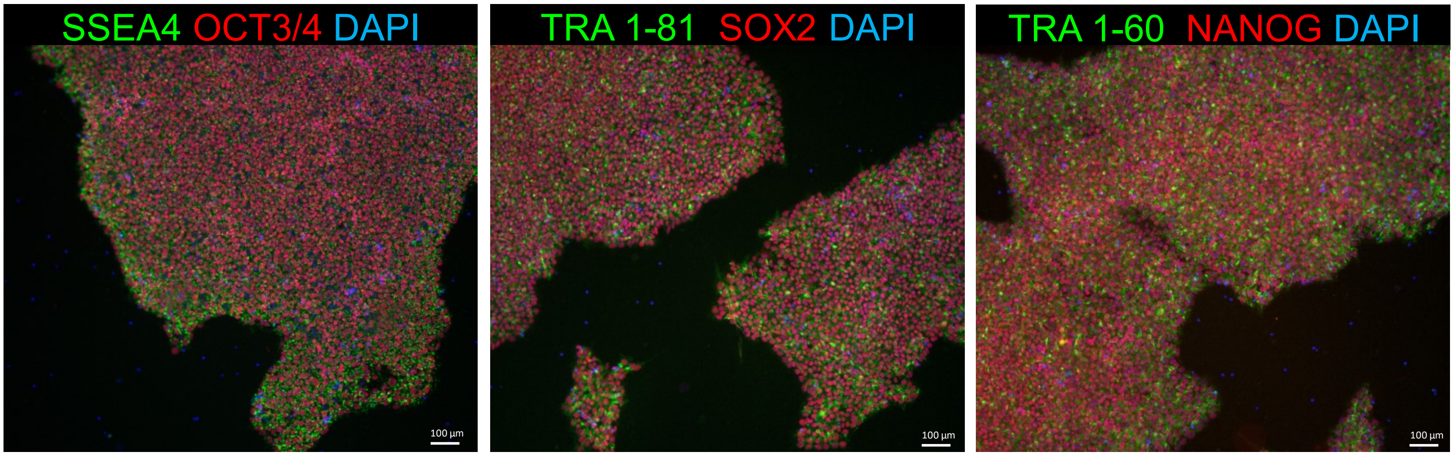

C. Pluritest

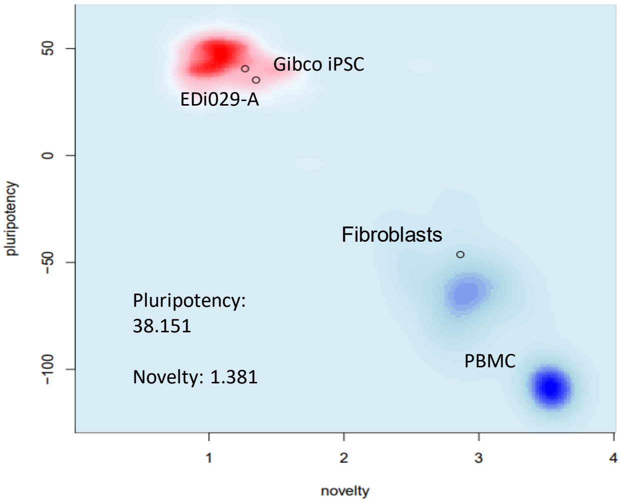

D. G-Band karyotype

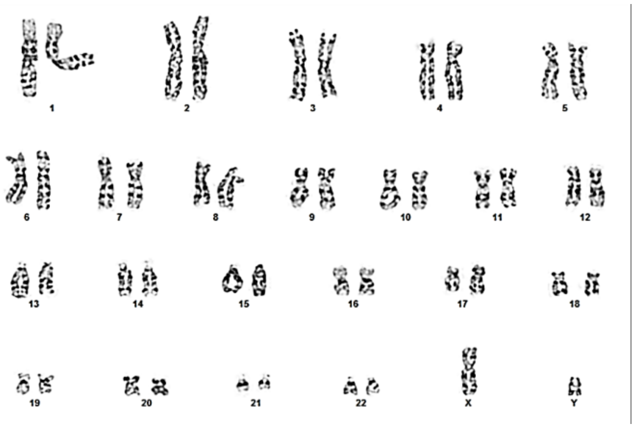

E. hPSC Scorecard

iPSCs				Embryoid Bodies			
Self-renew	Ecto	Meso	Endo	Self-renew	Ecto	Meso	Endo
+	−	−	−	−	+	+	+
0.75	0.33	0.37	-0.81	-5.16	2.13	3.91	1.96

F. Morphology

4 days post-thaw

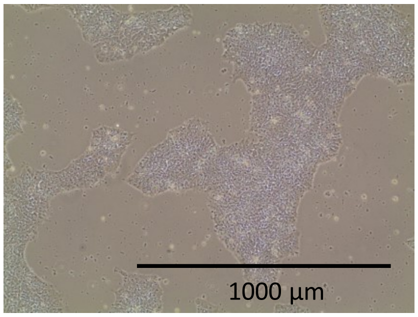

sFig.9: Characterization for iPSC line EDi030-A

A. AP

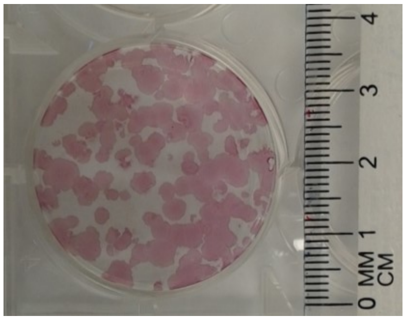

B. Immunocytochemistry

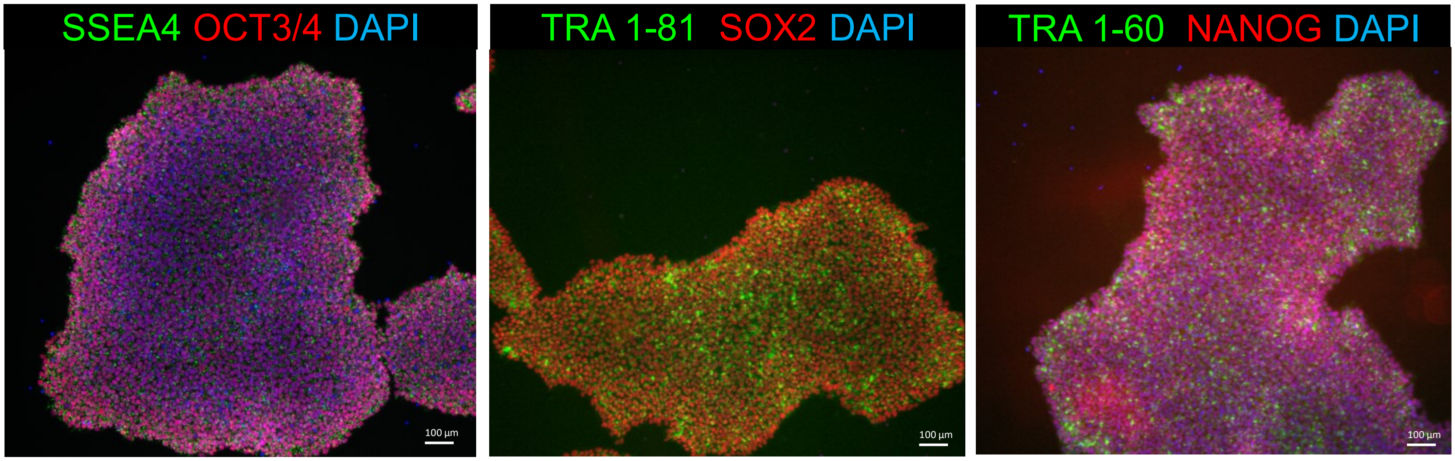

C. Pluritest

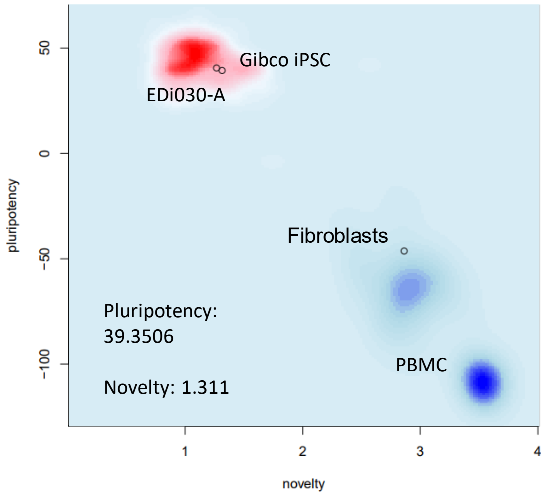

D. G-Band karyotype

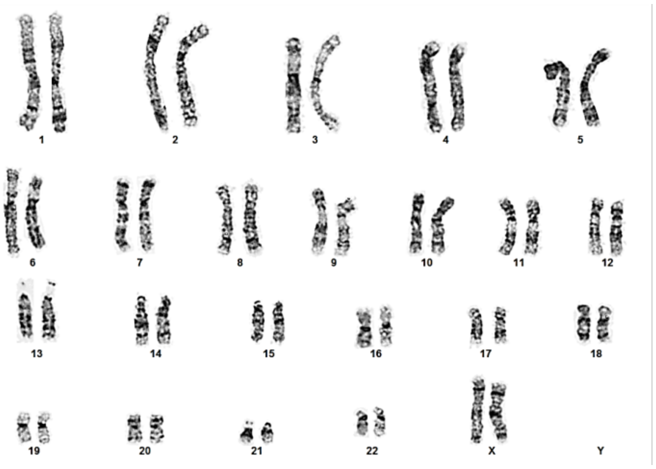

E. hPSC Scorecard

iPSCs				Embryoid Bodies			
Self-renew	Ecto	Meso	Endo	Self-renew	Ecto	Meso	Endo
+	−	−	−	−	+	+	+
-0.30	0.07	0.54	-1.48	-5.34	1.72	5.14	1.95

F. Morphology

2 days post-thaw

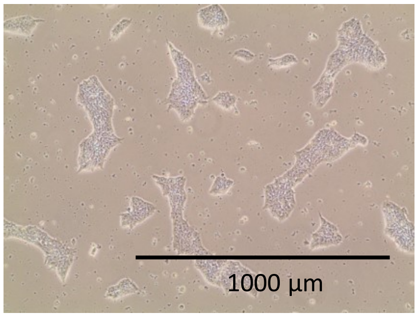

sFig.10: Characterization for iPSC line EDi031-A

A. AP

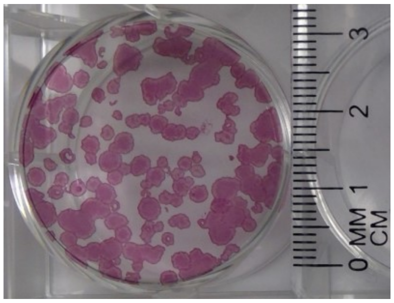

B. Immunocytochemistry

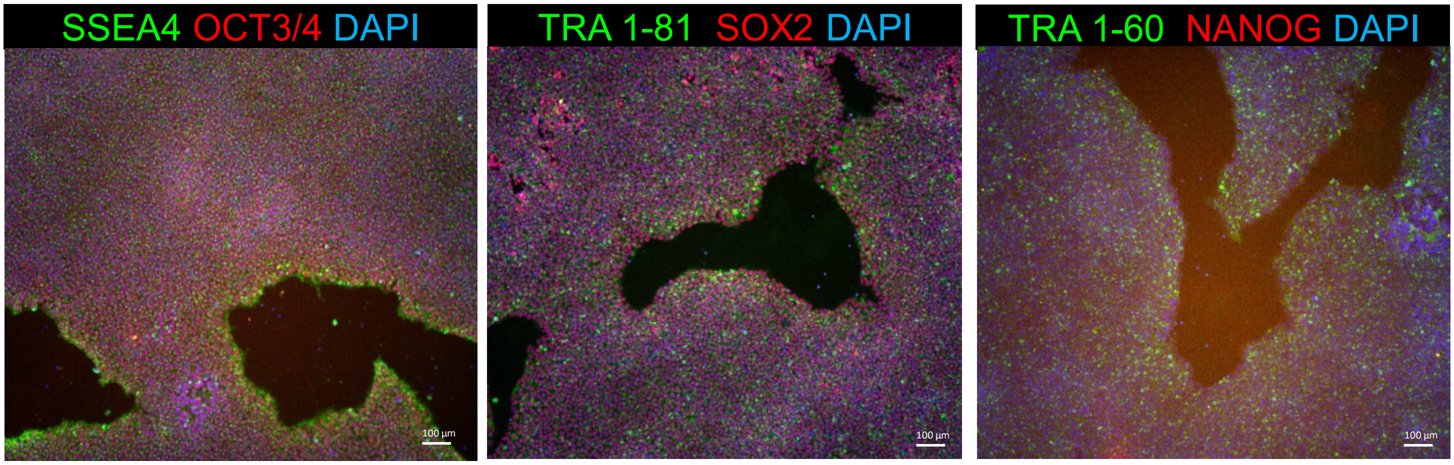

C. Pluritest

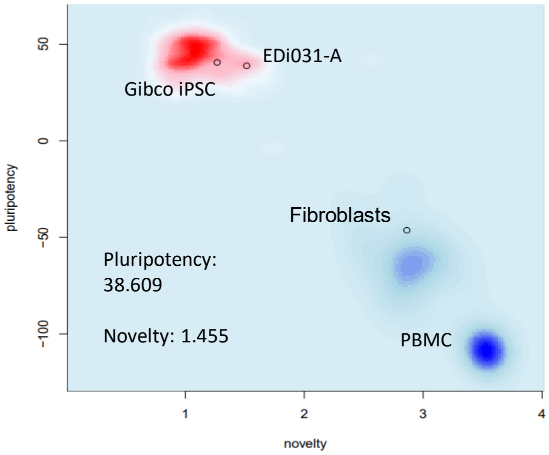

D. G-Band karyotype

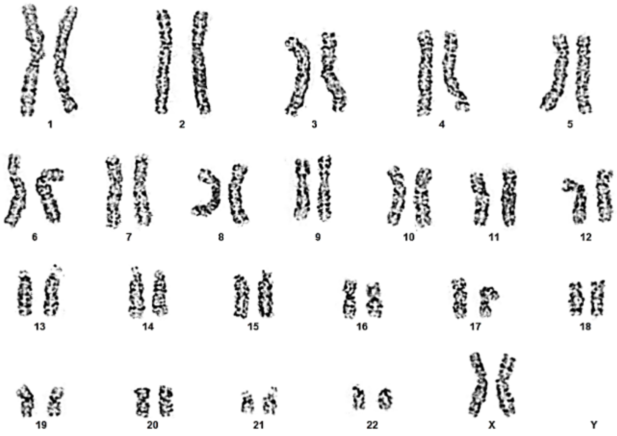

E. hPSC Scorecard

iPSCs				Embryoid Bodies			
Self-renew	Ecto	Meso	Endo	Self-renew	Ecto	Meso	Endo
-0.19	-0.46	-0.24	-1.30	-6.33	1.77	2.65	1.65

F. Morphology

2 days post-thaw

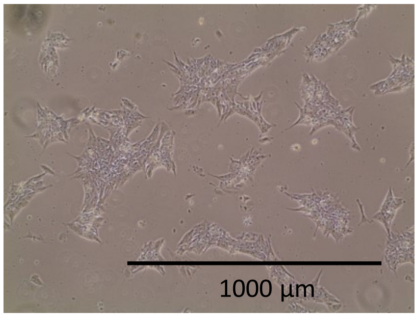

sFig.11: Characterization for iPSC line EDi032-A

A. AP

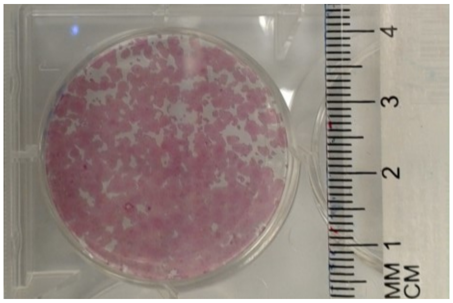

B. Immunocytochemistry

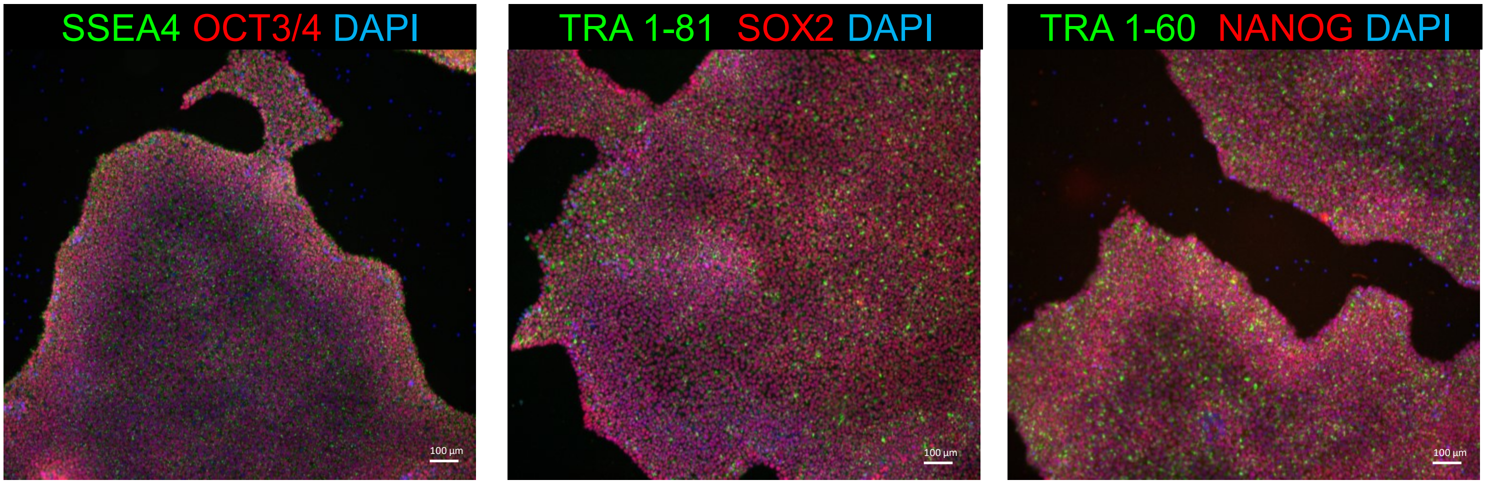

C. Pluritest

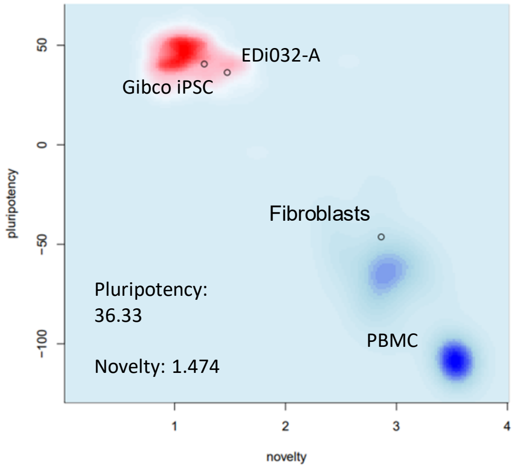

D. G -Band karyotype

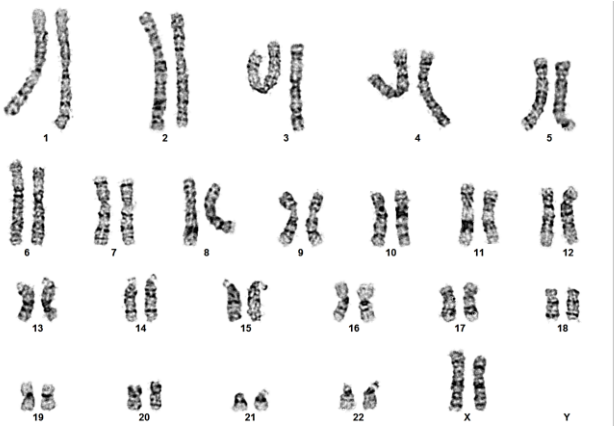

E. hPSC Scorecard

iPSCs				Embryoid Bodies			
Self-renew	Ecto	Meso	Endo	Self-renew	Ecto	Meso	Endo
+	−	−	−	−	+	+	+
0.24	-0.06	0.08	-1.36	-6.85	1.85	2.34	0.78

F. Morphology

4 days post-thaw

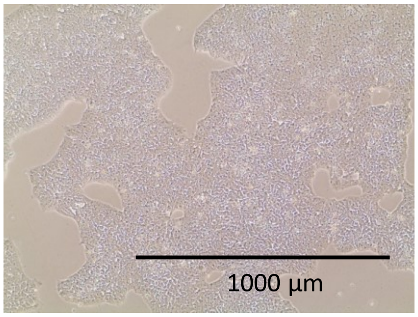

sFig.12: Characterization for iPSC line EDi033-A

A. AP

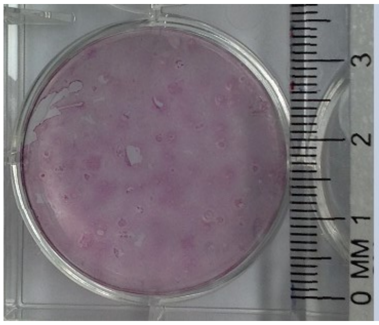

B. Immunocytochemistry

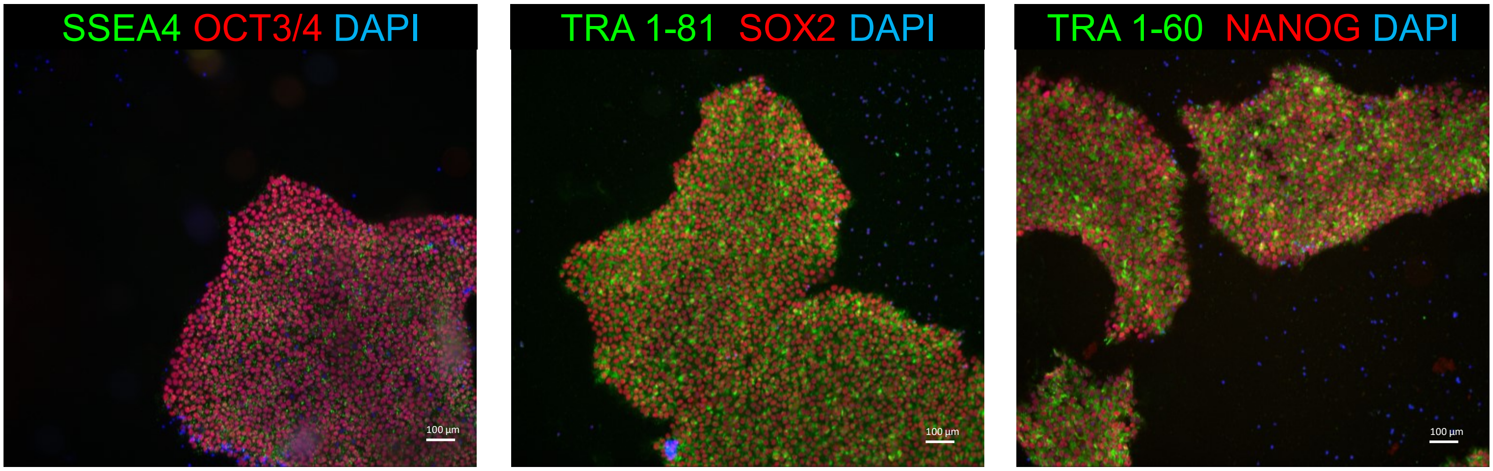

C. Pluritest

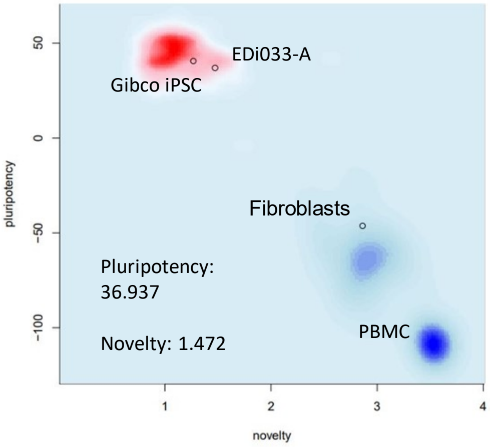

D. G -Band karyotype

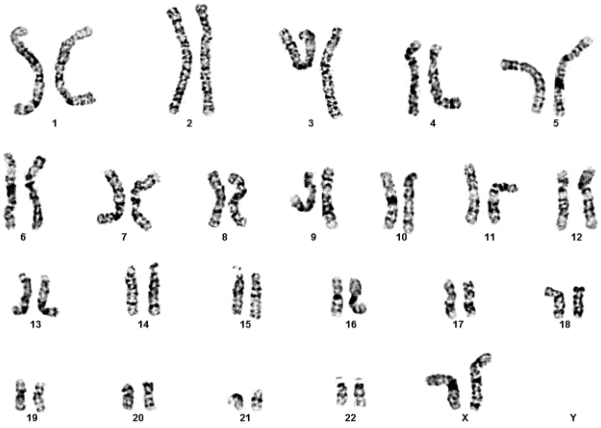

E. hPSC Scorecard

iPSCs				Embryoid Bodies			
Self-renew	Ecto	Meso	Endo	Self-renew	Ecto	Meso	Endo
+	−	−	−	−	+	+	+
0.23	-0.02	-0.18	-1.03	-3.58	1.77	3.93	1.85

F. Morphology

7 days post-thaw

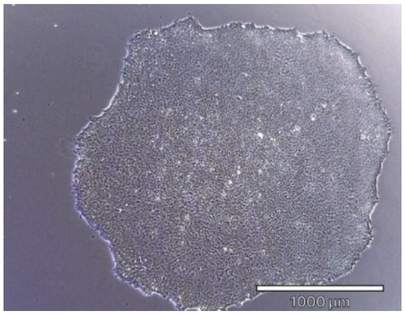

sFig.13: Characterization for iPSC line EDi034-A

A. AP

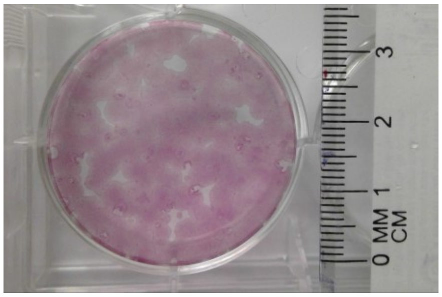

B. Immunocytochemistry

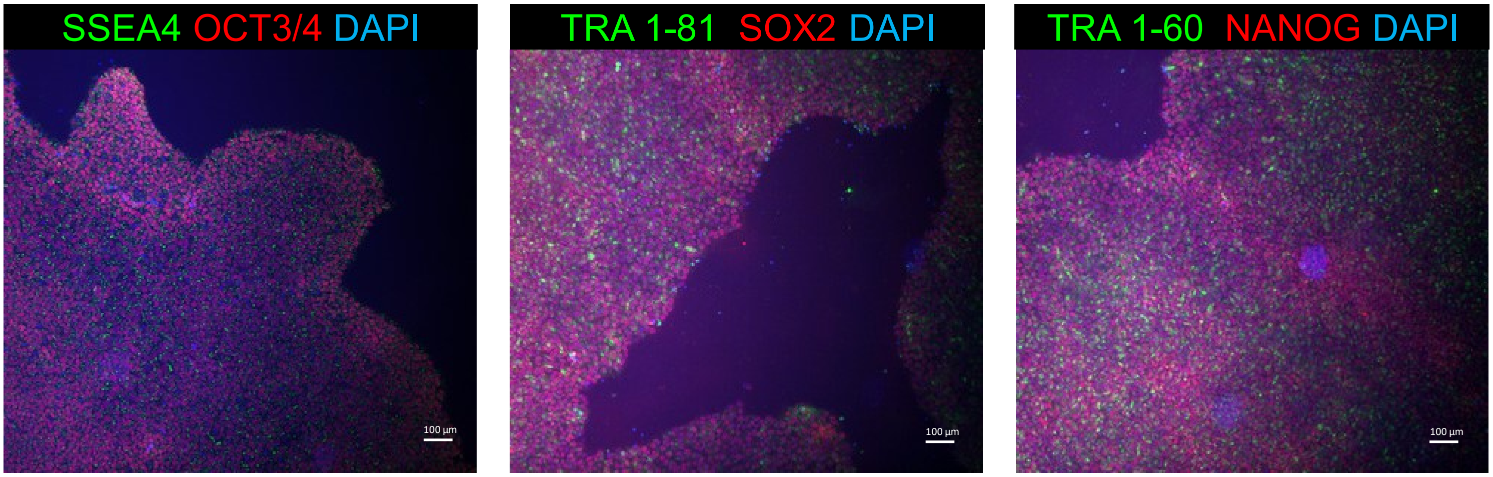

C. Pluritest

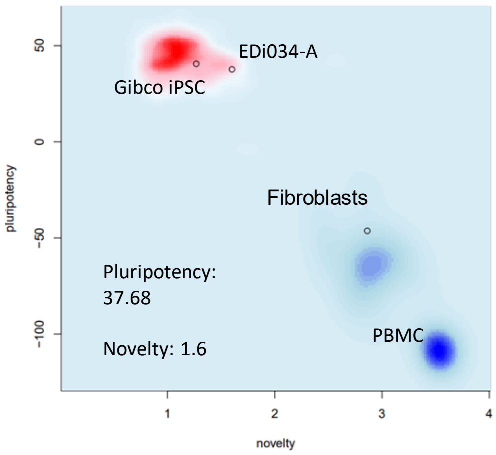

D. G -Band karyotype

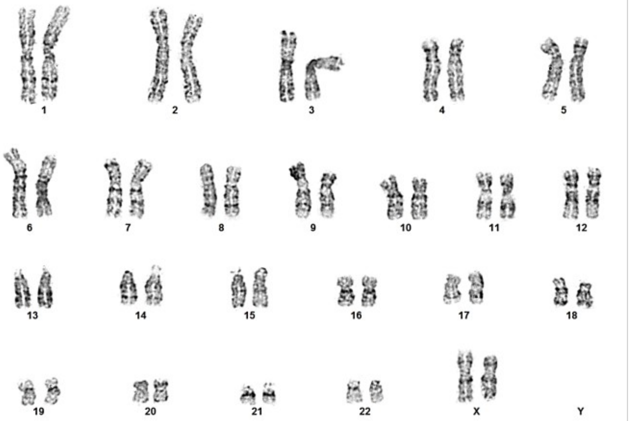

E. hPSC Scorecard

iPSCs				Embryoid Bodies			
Self-renew	Ecto	Meso	Endo	Self-renew	Ecto	Meso	Endo
+	−	−	−	−	+	+	+
0.77	-0.25	-0.22	-1.57	-4.24	2.56	3.43	1.67

F. Morphology

2 days post-thaw

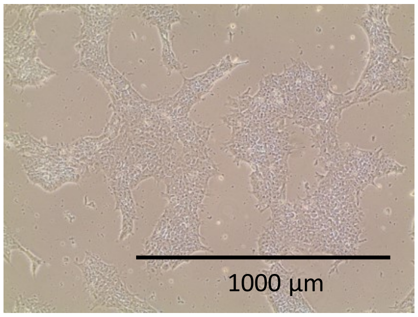

sFig.14: Characterization for iPSC line EDi035-A

A. AP

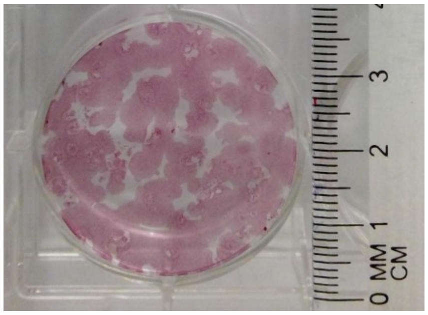

B. Immunocytochemistry

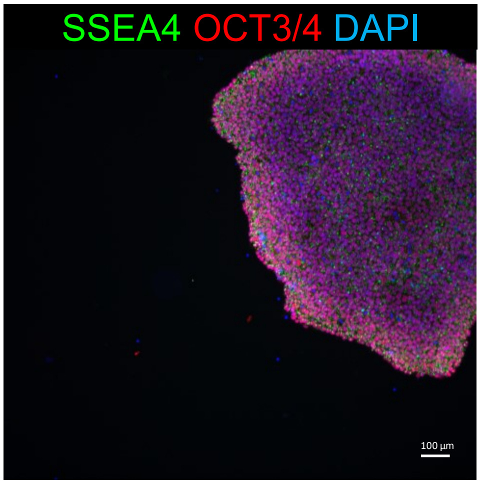

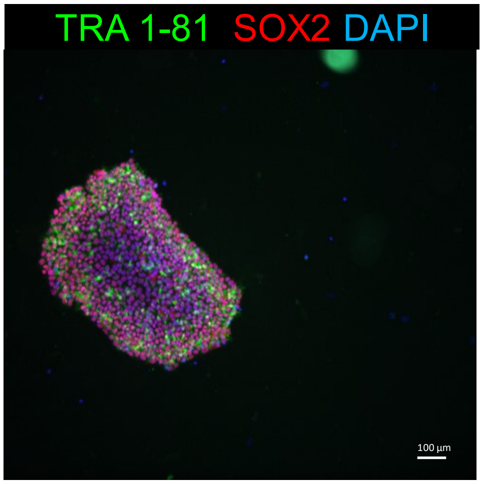

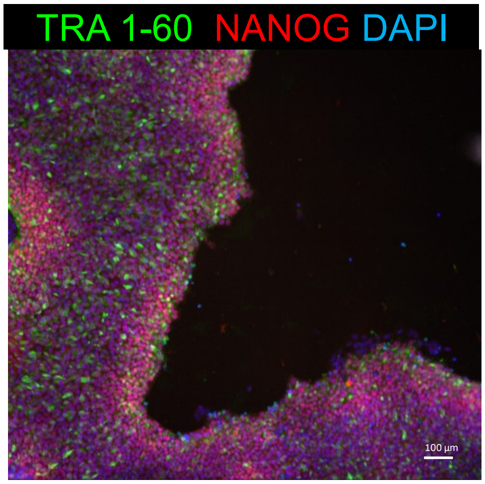

C. Pluritest

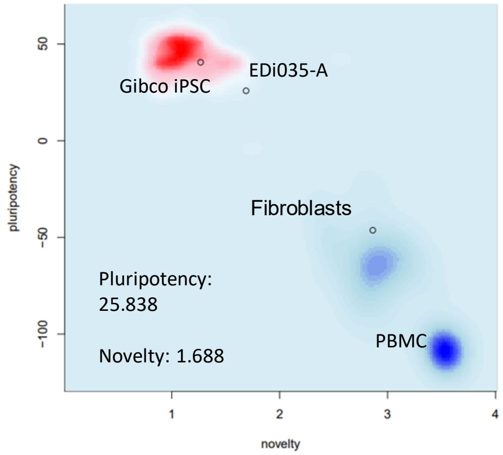

D. G -Band karyotype

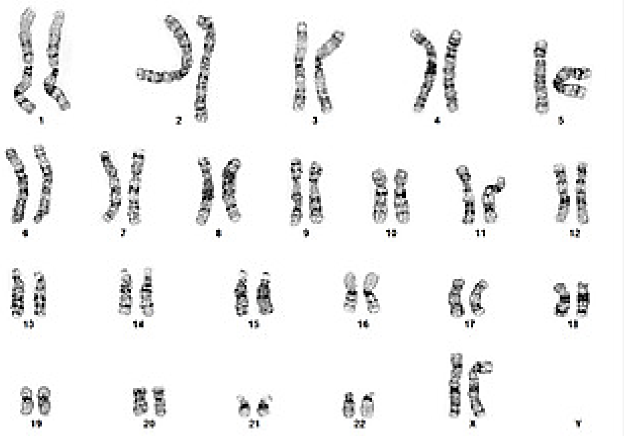

E. hPSC Scorecard

iPSCs				Embryoid Bodies			
Self-renew	Ecto	Meso	Endo	Self-renew	Ecto	Meso	Endo
0.39	0.19	0.02	-1.22	-6.69	1.81	0.64	0.29

F. Morphology

7 days post-thaw

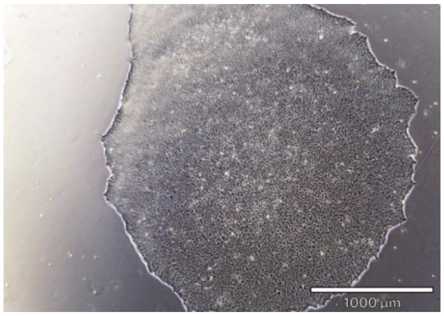

sFig.15: Characterization for iPSC line EDi036-A

A. AP

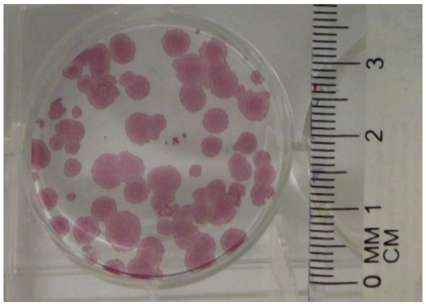

B. Immunocytochemistry

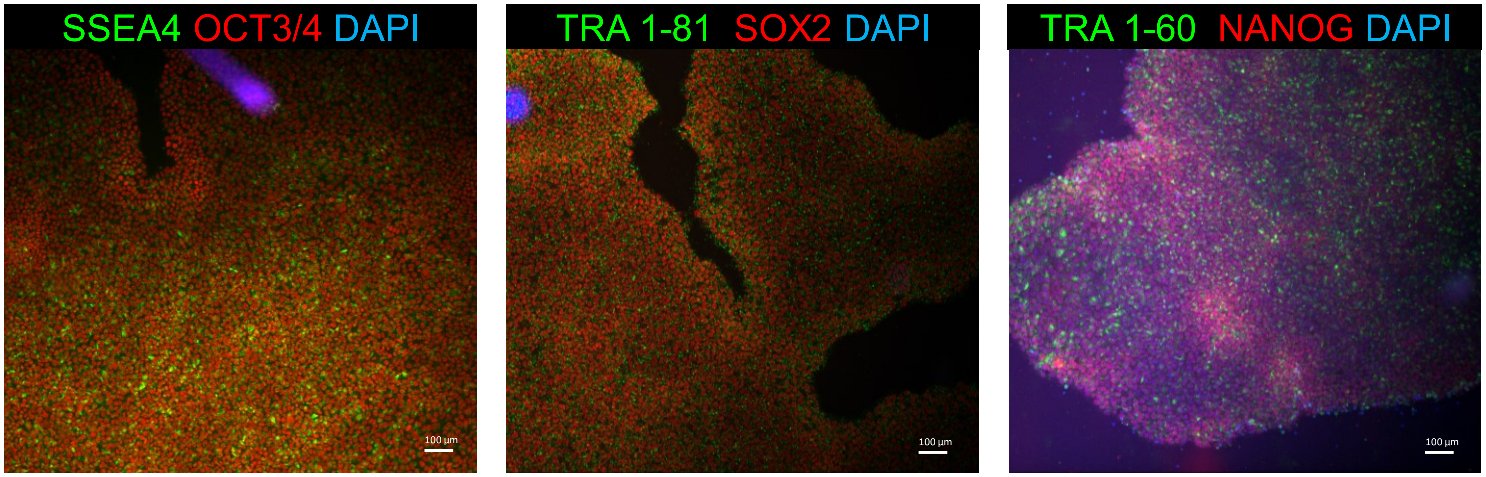

C. Pluritest

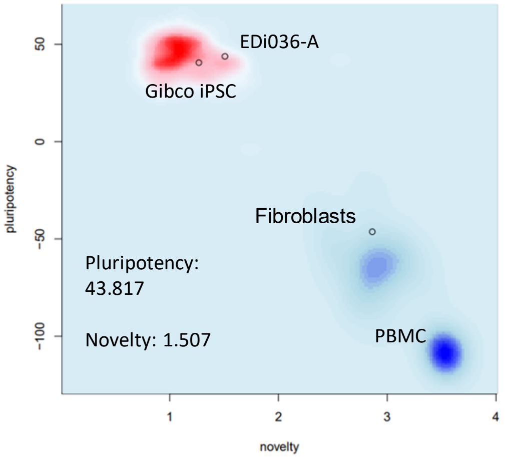

D. G -Band karyotype

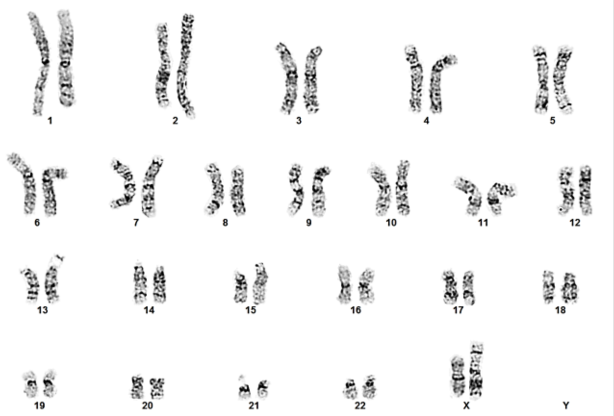

E. hPSC Scorecard

iPSCs				Embryoid Bodies			
Self-renew	Ecto	Meso	Endo	Self-renew	Ecto	Meso	Endo
+	−	−	−	−	+	+	+
0.50	0.37	0.20	-1.03	-6.35	1.90	1.61	0.66

F. Morphology

2 days post-thaw

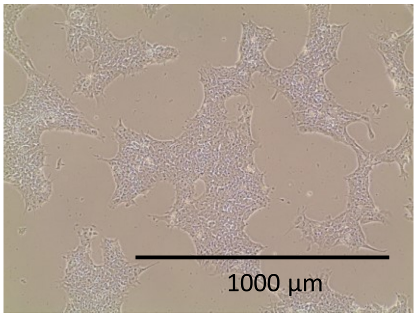

sFig.16: Characterization for iPSC line EDi037-A

A. AP

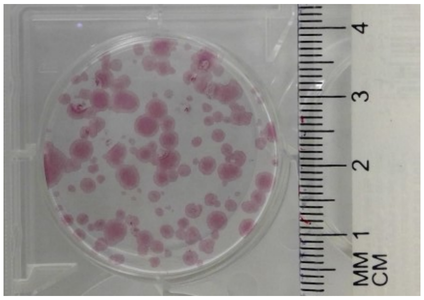

B. Immunocytochemistry

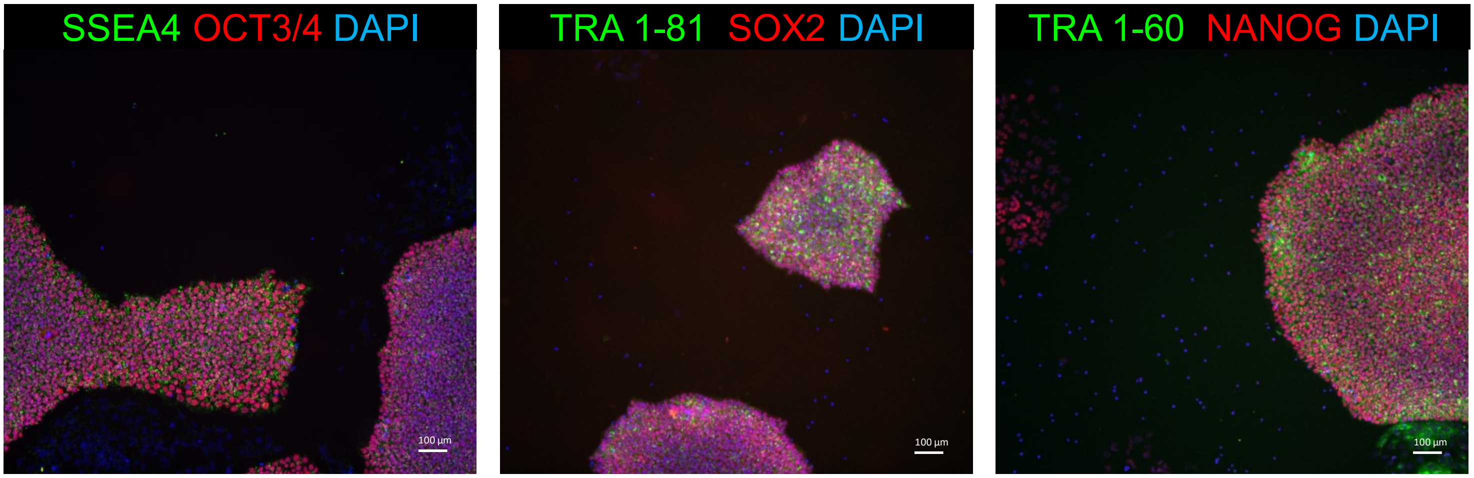

C. Pluritest

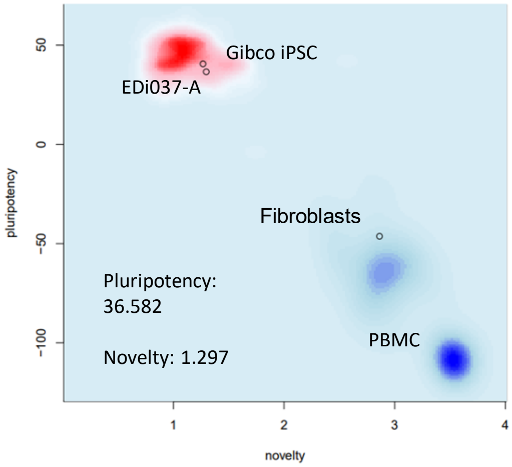

D. G -Band karyotype

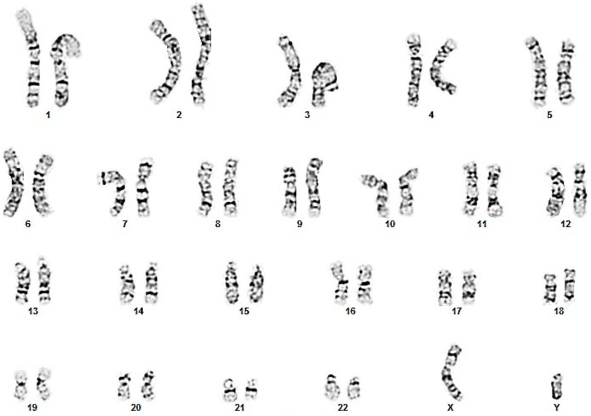

E. hPSC Scorecard

iPSCs				Embryoid Bodies			
Self-renew	Ecto	Meso	Endo	Self-renew	Ecto	Meso	Endo
+	−	−	−	−	+	+	+
0.05	0.20	0.05	-0.75	-7.38	1.65	1.80	0.87

F. Morphology

2 days post-thaw

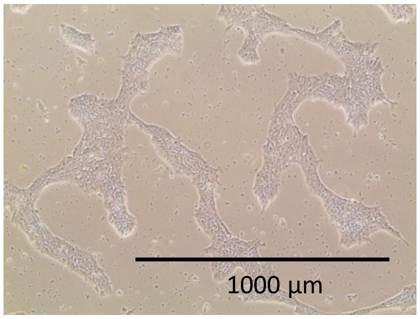

sFig.17: Characterization for iPSC line EDi038-A

A. AP

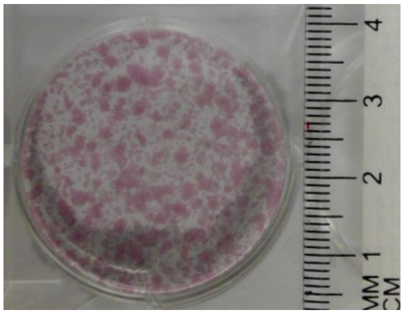

B. Immunocytochemistry

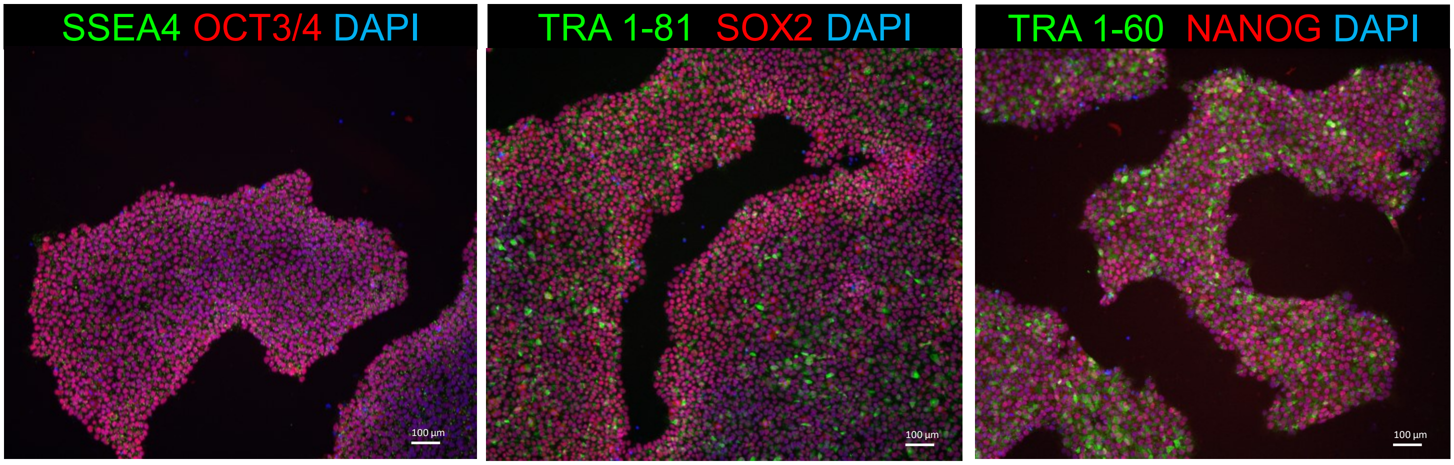

C. Pluritest

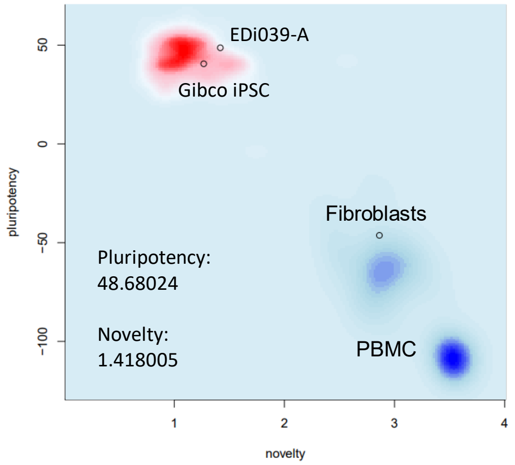

D. G-Band karyotype

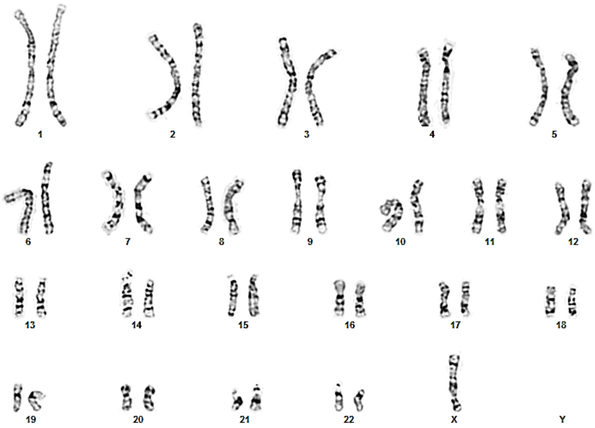

E. hPSC Scorecard

iPSCs				Embryoid Bodies			
Self-renew	Ecto	Meso	Endo	Self-renew	Ecto	Meso	Endo
+	−	−	−	−	+	+	+
0.61	-0.09	0.48	-1.10	-6.22	1.41	6.00	1.73

F. Morphology

11 days post-thaw

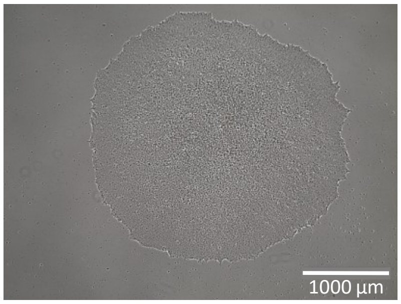

sFig.18: Characterization for iPSC line EDi039-A

A. AP

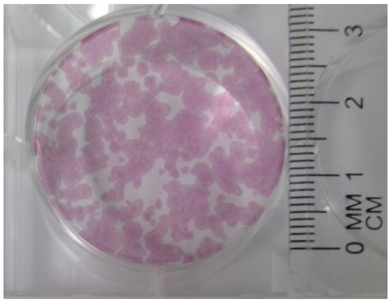

B. Immunocytochemistry

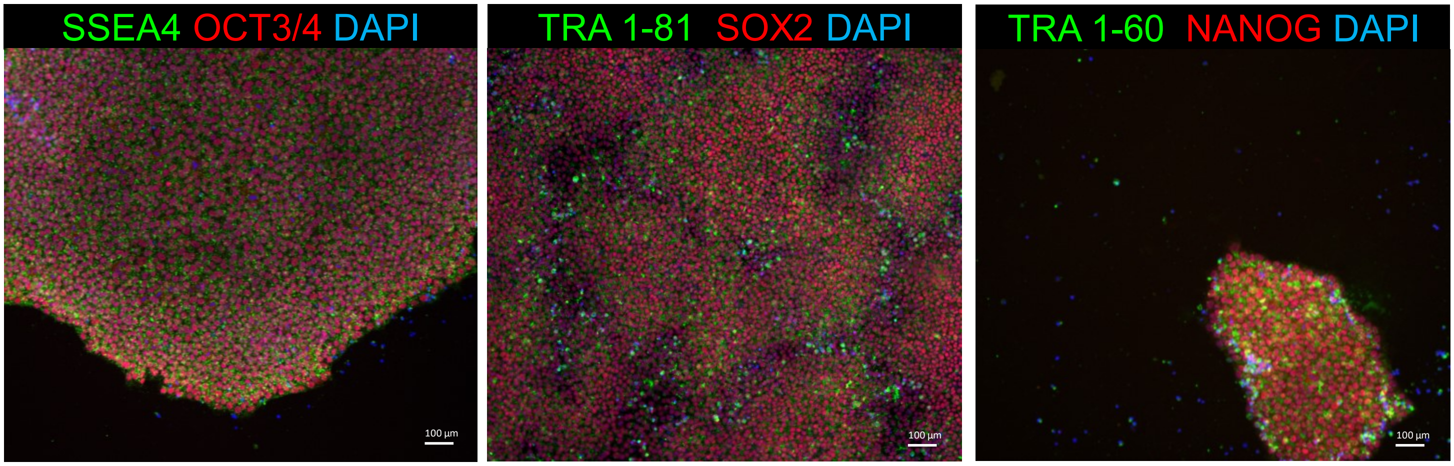

C. PluriTest

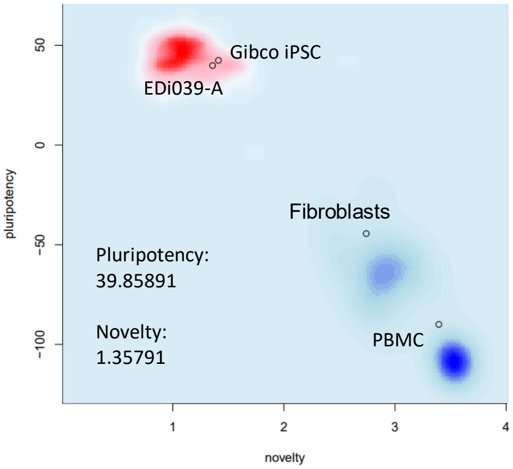

D. G-Band karyotype

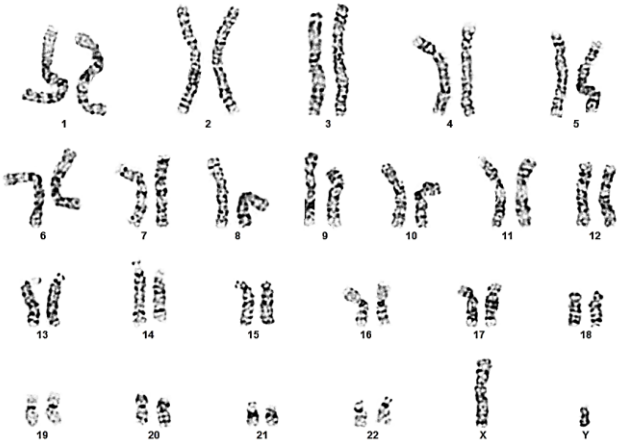

E. hPSC Scorecard

iPSCs				Embryoid Bodies			
Self-renew	Ecto	Meso	Endo	Self-renew	Ecto	Meso	Endo
+	−	−	−	−	+	+	+
-0.19	0.14	-0.15	-1.05	-6.74	2.08	1.07	0.64

F. Morphology

11 days post-thaw

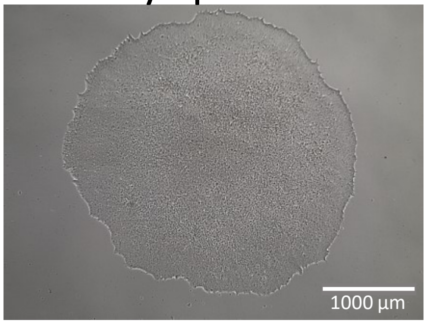

sFig.19: Characterization for iPSC line EDi040-A

A. AP

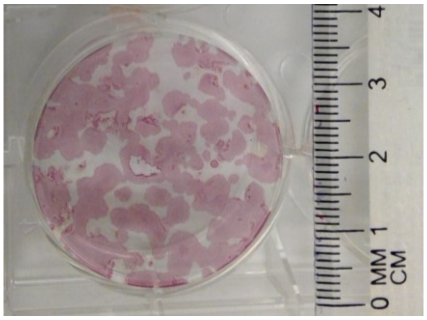

B. Immunocytochemistry

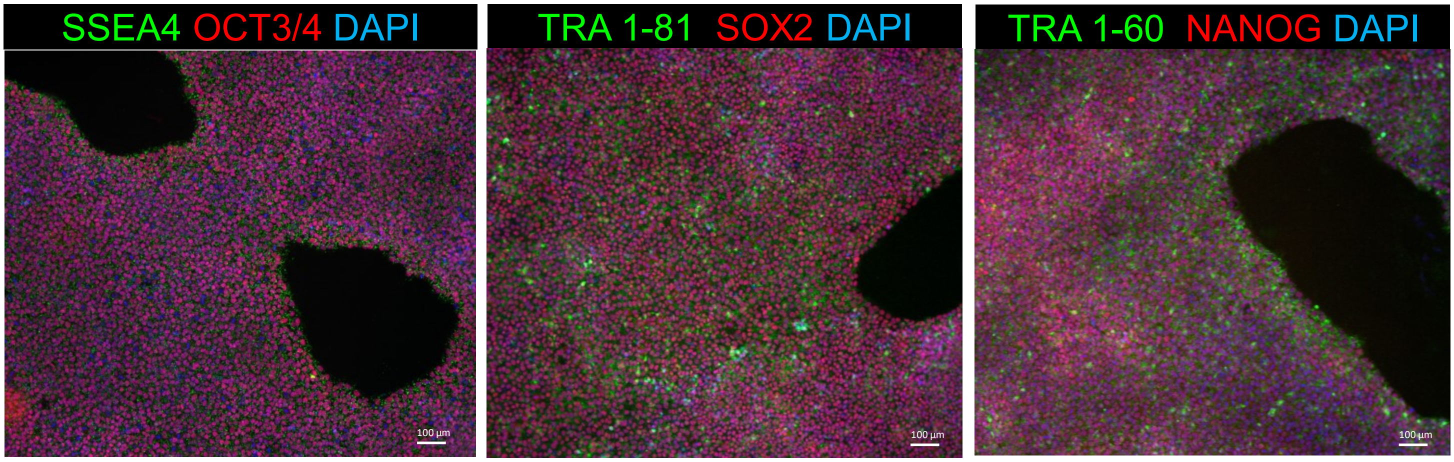

C. Pluritest

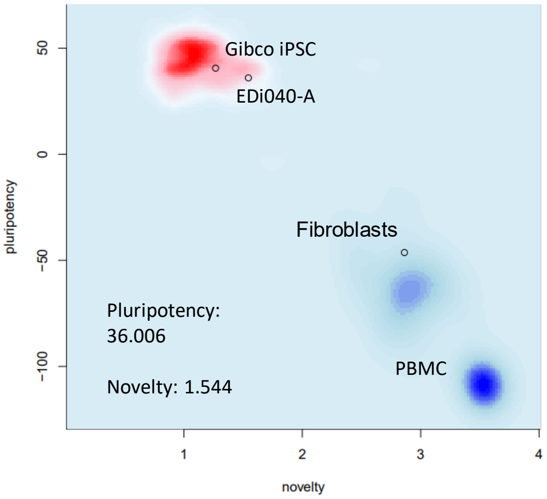

D. G-Band karyotype

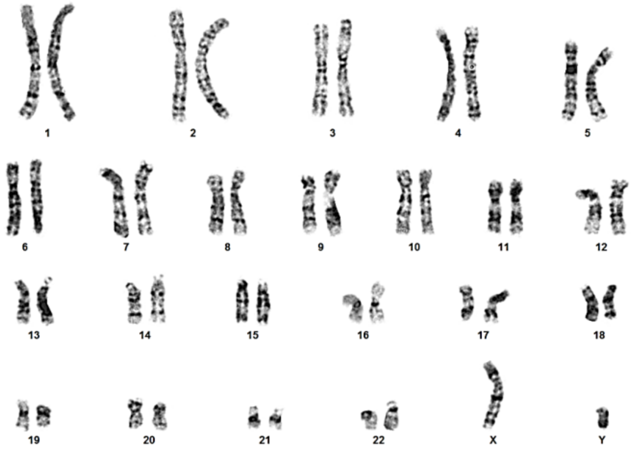

E. hPSC Scorecard

iPSCs				Embryoid Bodies			
Self-renew	Ecto	Meso	Endo	Self-renew	Ecto	Meso	Endo
+	−	−	−	−	+	+	+
0.01	-0.45	0.24	-0.94	-3.18	1.57	0.98	-0.56

F. Morphology

2 days post-thaw

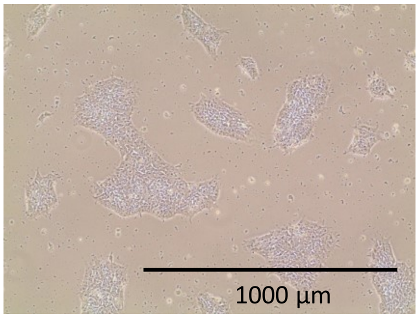

sFig.20: Characterization for iPSC line EDi041-A

A. AP

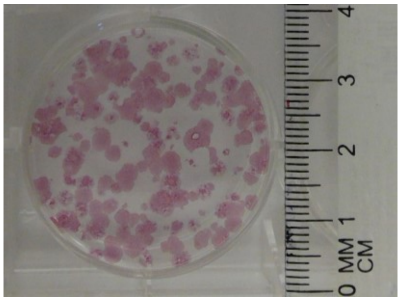

B. Immunocytochemistry

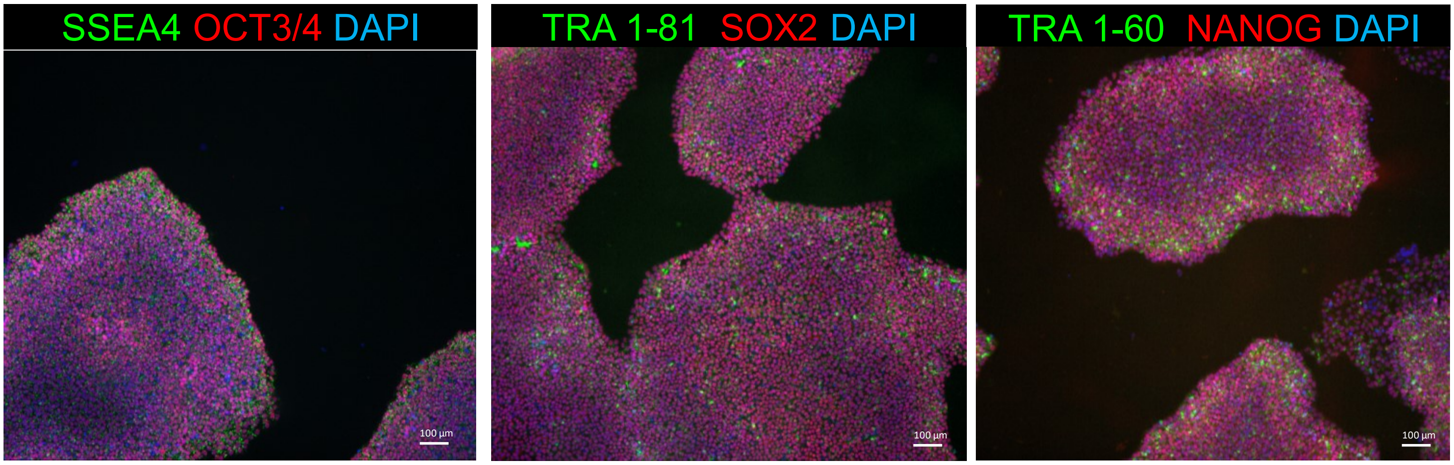

C. Pluritest

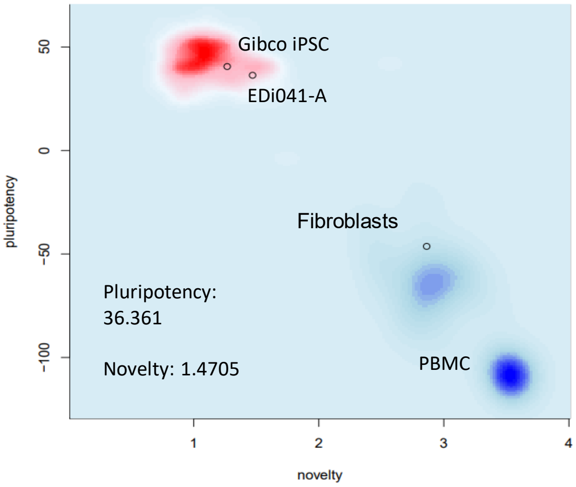

D. G-Band karyotype

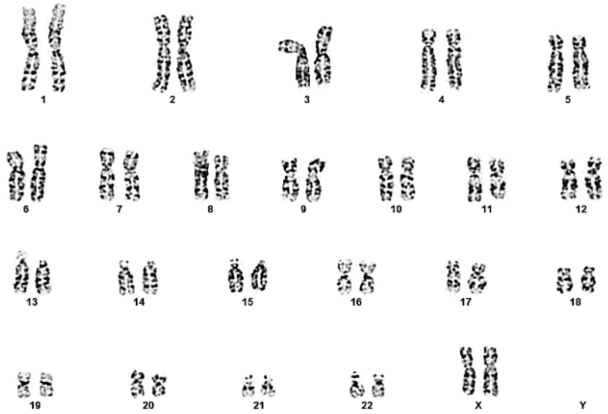

E. hPSC Scorecard

iPSCs				Embryoid Bodies			
Self-renew	Ecto	Meso	Endo	Self-renew	Ecto	Meso	Endo
+	−	−	−	−	+	+	+
-0.18	0.05	-0.32	-1.24	-7.04	1.61	1.42	0.67

F. Morphology

3 days post-thaw

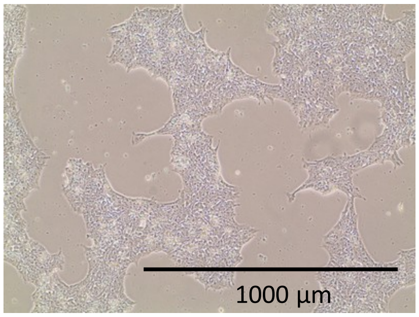

sFig.21: Characterization for iPSC line EDi042-A

A. AP

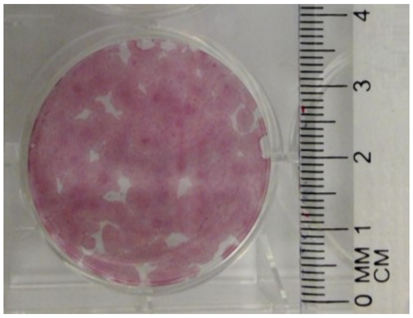

B. Immunocytochemistry

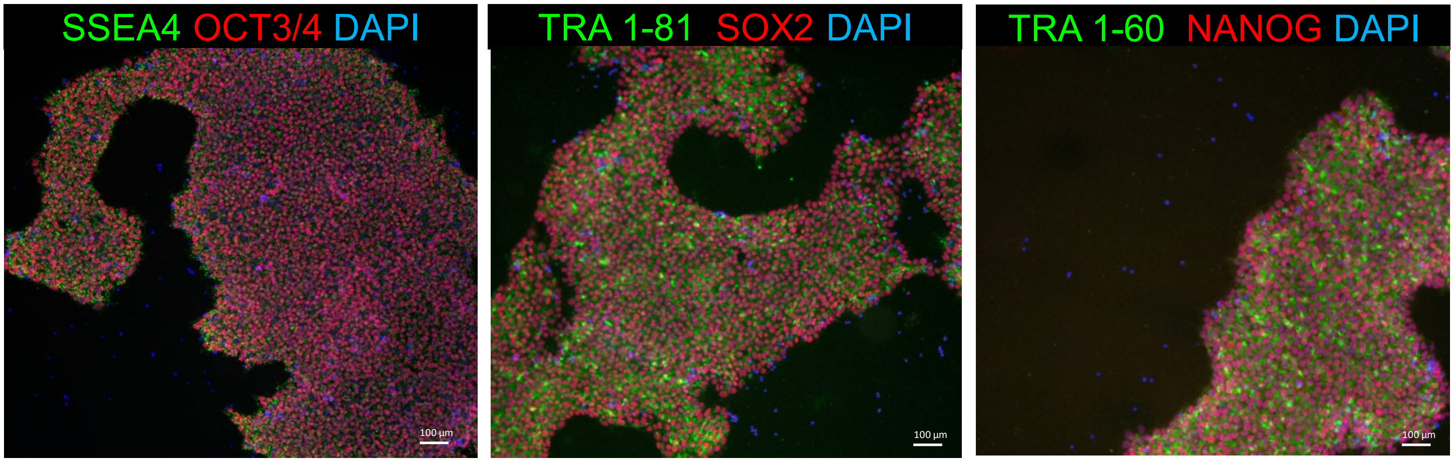

C. Pluritest

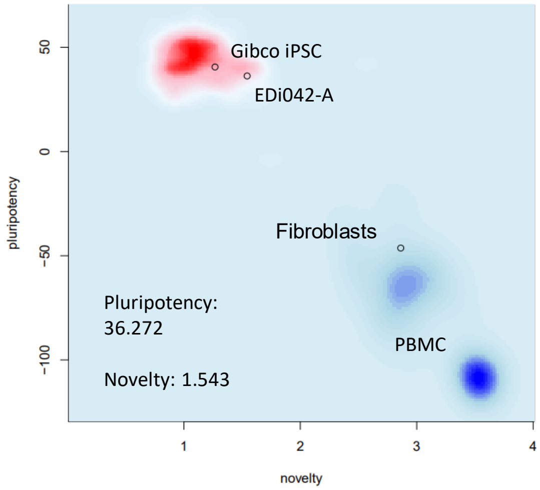

D. G-Band karyotype

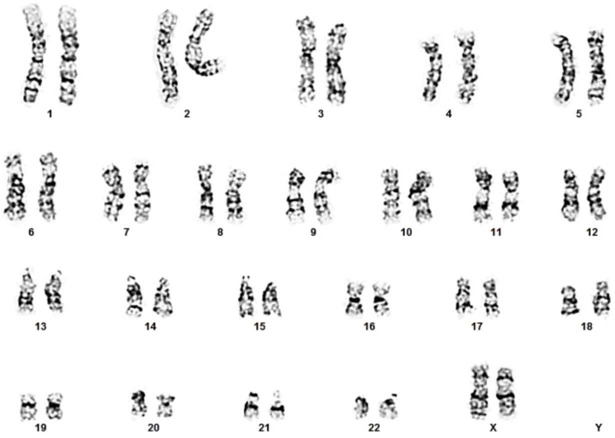

E. hPSC Scorecard

iPSCs				Embryoid Bodies			
Self-renew	Ecto	Meso	Endo	Self-renew	Ecto	Meso	Endo
+	−	−	−	−	+	+	−
0.05	-0.13	0.05	-1.04	-5.15	2.62	1.07	0.22

F. Morphology

4 days post-thaw

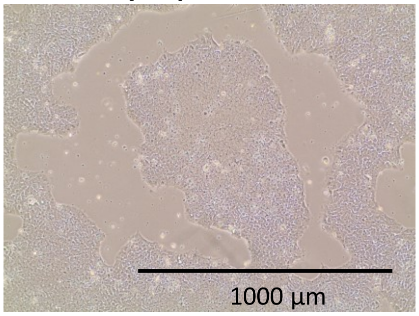

sFig.22: Characterization for iPSC line EDi043-A

A. AP

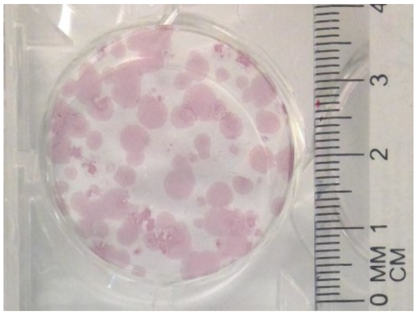

B. Immunocytochemistry

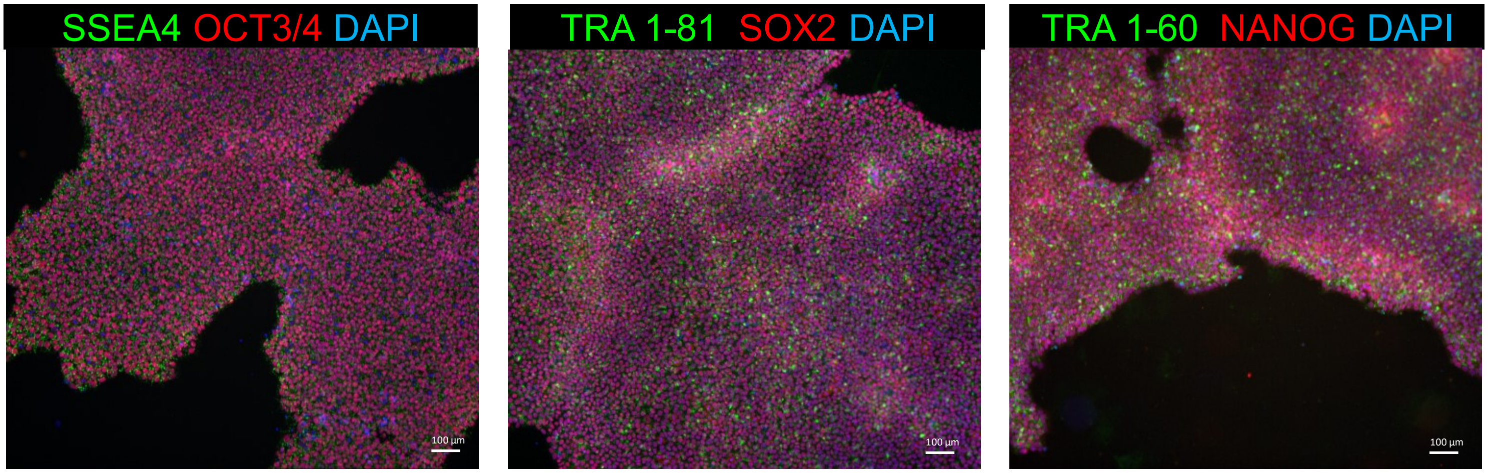

C. Pluritest

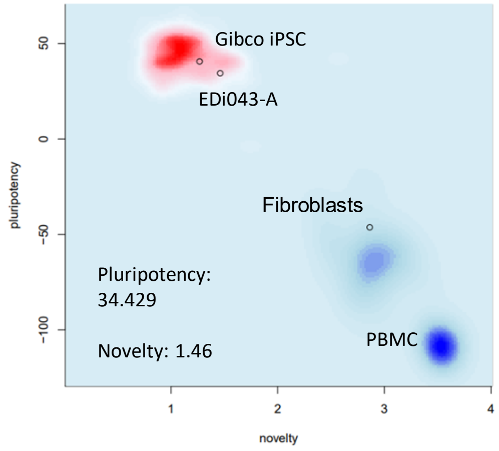

D. G -Band karyotype

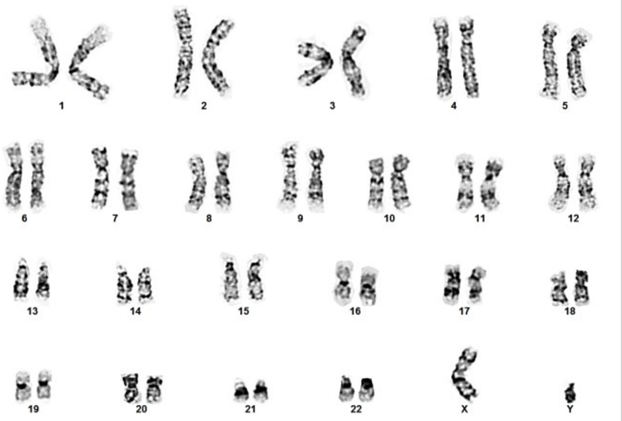

E. hPSC Scorecard

iPSCs				Embryoid Bodies			
Self-renew	Ecto	Meso	Endo	Self-renew	Ecto	Meso	Endo
+	−	−	−	−	+	+	+
-0.03	0.38	0.07	-1.23	-6.46	1.59	2.57	1.19

F. Morphology

7 days post-thaw

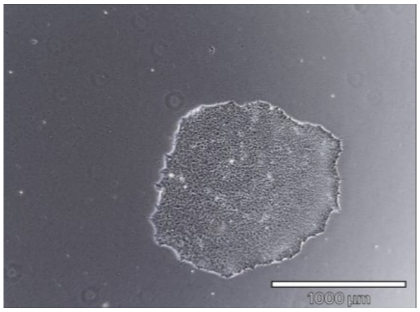

sFig.23: Characterization for iPSC line EDi044-A

A. AP

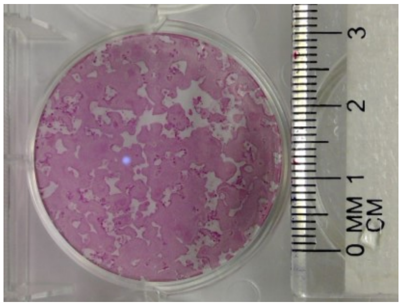

B. Immunocytochemistry

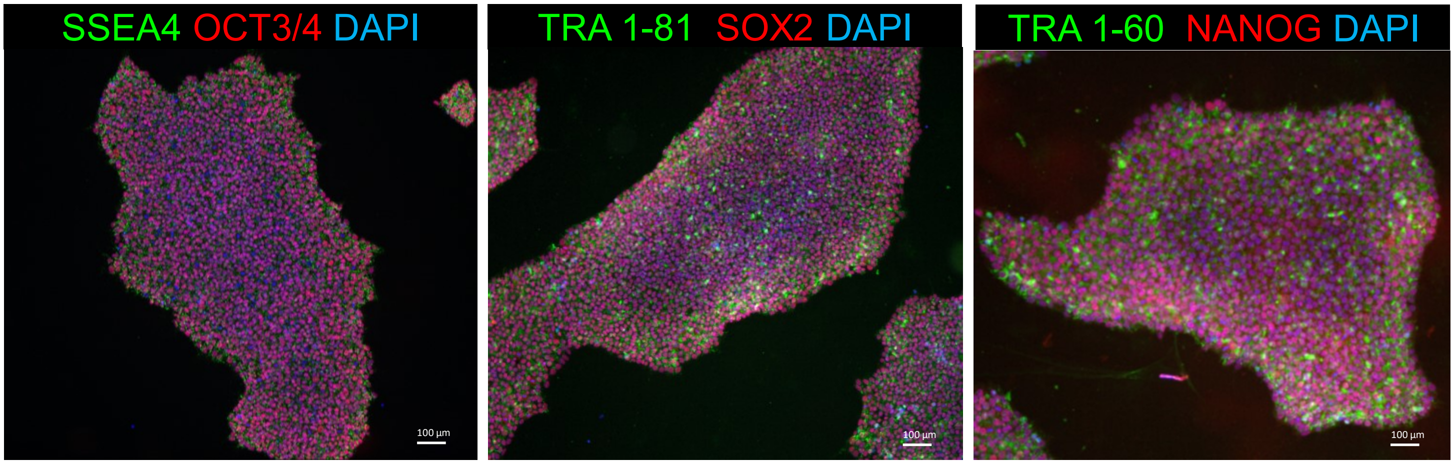

C. Pluritest

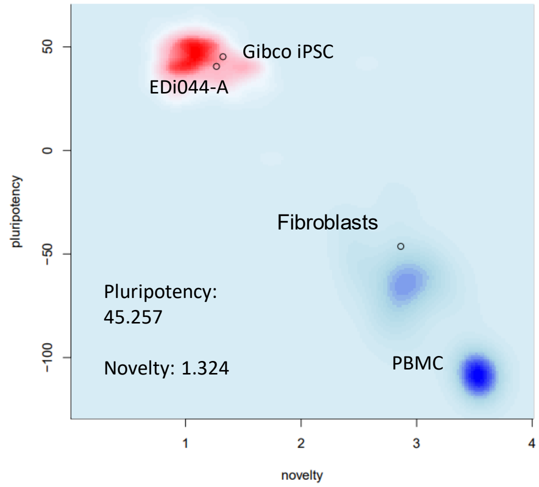

D. G-Band karyotype

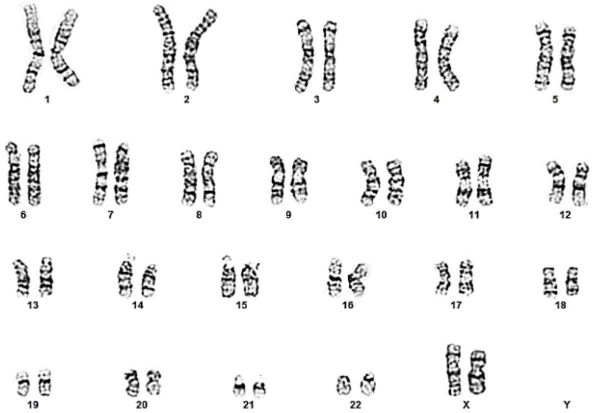

E. hPSC Scorecard

iPSCs				Embryoid Bodies			
Self-renew	Ecto	Meso	Endo	Self-renew	Ecto	Meso	Endo
-0.75	-0.19	0.58	-0.80	-6.29	1.65	3.42	0.94

F. Morphology

4 days post-thaw

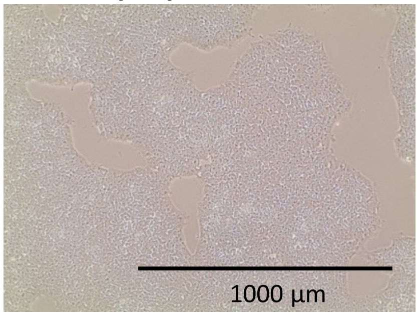

sFig.24: Characterization for iPSC line EDi045-A

A. AP

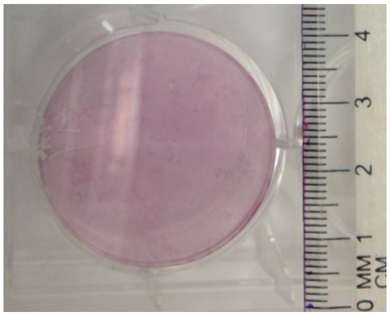

B. Immunocytochemistry

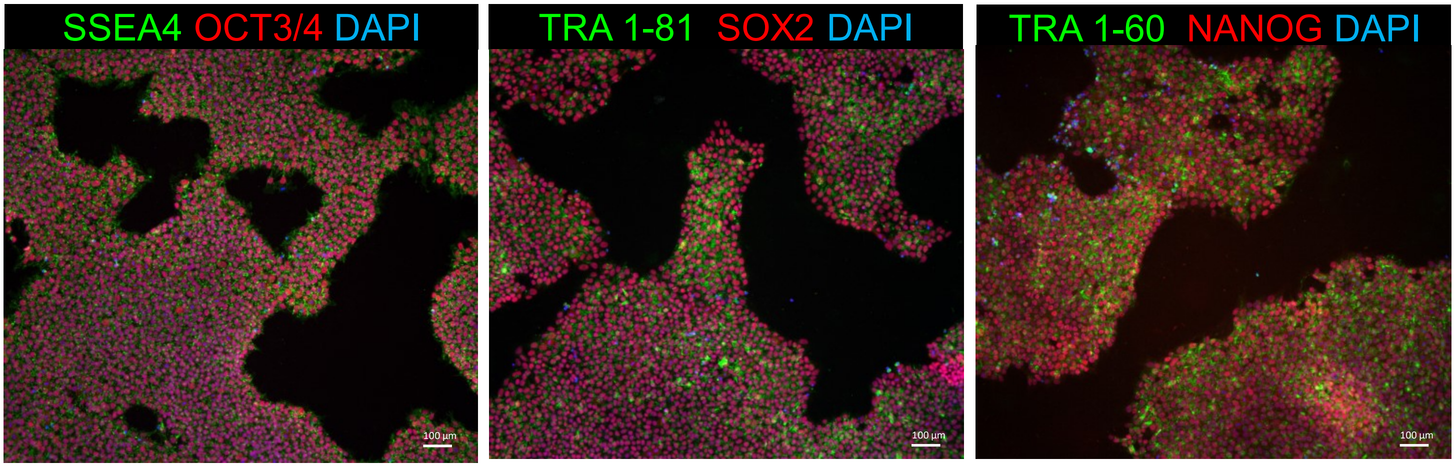

C. Pluritest

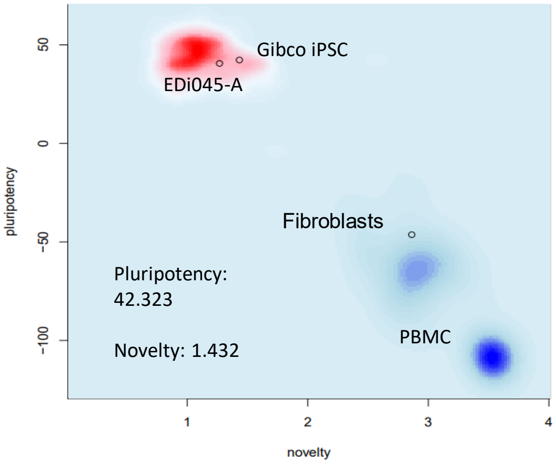

D. G-Band karyotype

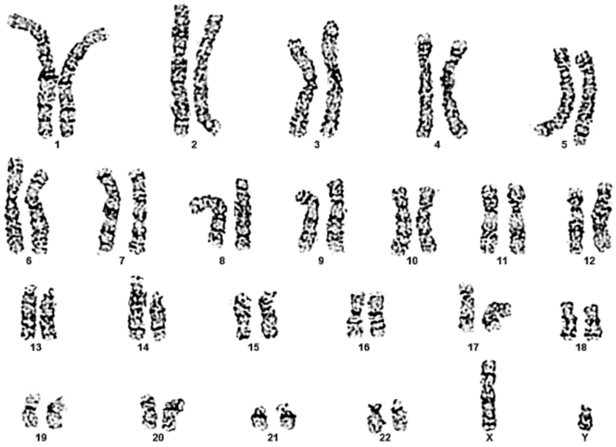

E. hPSC Scorecard

iPSCs				Embryoid Bodies			
Self-renew	Ecto	Meso	Endo	Self-renew	Ecto	Meso	Endo
+	−	−	−	−	+	+	+
-0.11	0.15	0.22	-0.81	-6.43	2.29	1.53	0.62

F. Morphology

14 days post-thaw

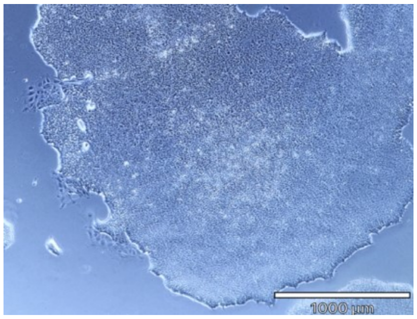

sFig.25: EBNA assay for LBC lines

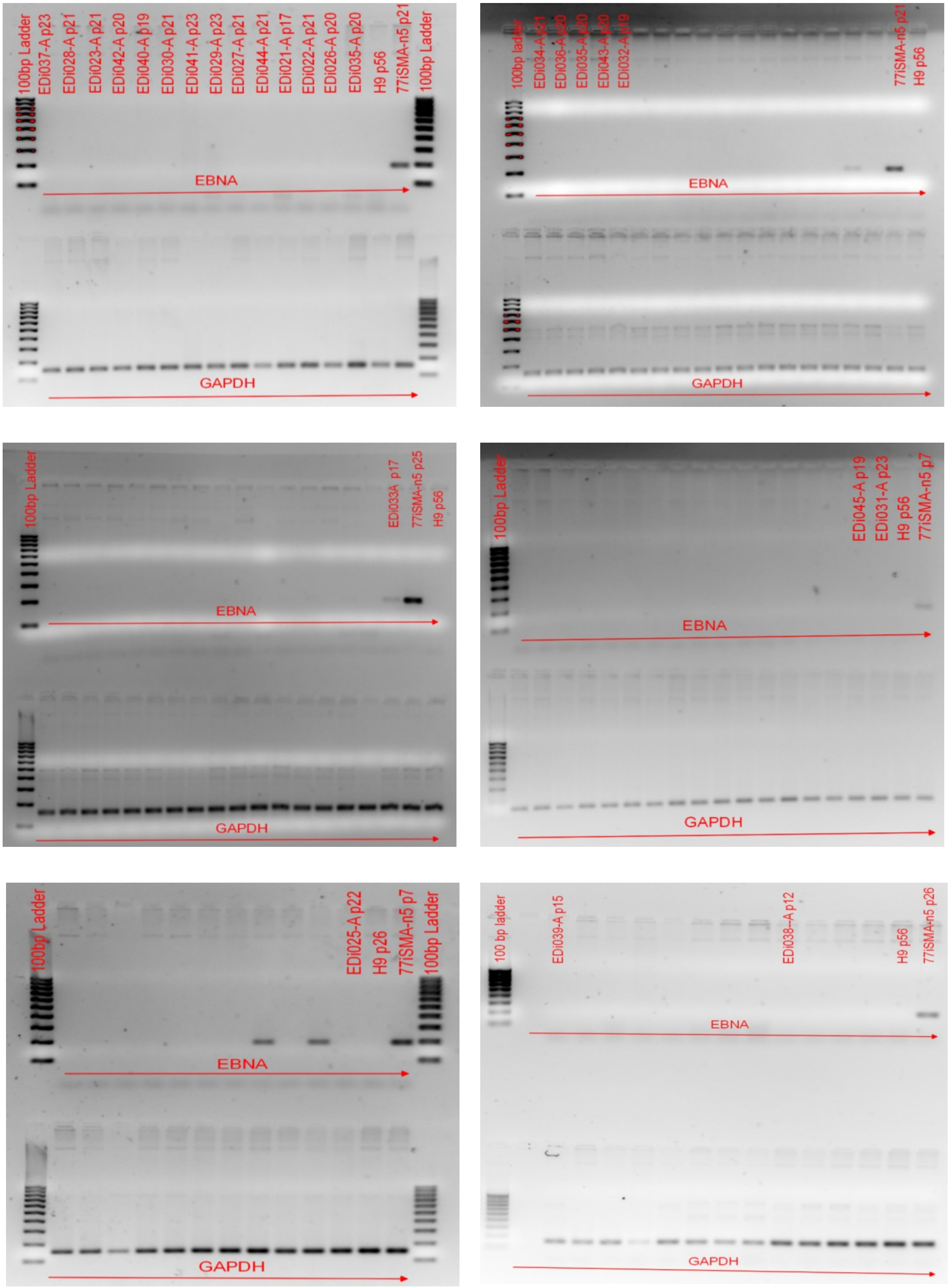

sFig25: 2% Agarose Gel images showing lack of EBNA persistence in all LBC iPSC lines. 77iSMA-n5 p21 = positive control iPSC for EBNA persistence. H9 p56 = Human ESC line H9, negative control for EBNA

sFig.26: T-cell Clonality

A) TCRB Tube A(240-285bp)
Target: Vß +Jß1/2

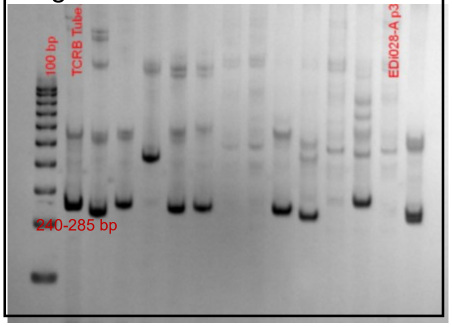

B) TCRB Tube B(240-285bp)
Target: Vß +Jß2

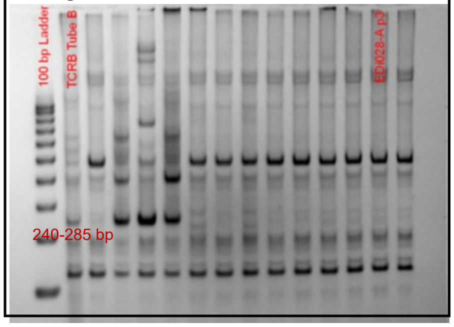

C) TCRB Tube C(170-210bp),
(285-325bp).Target: Dß +Jß1/2

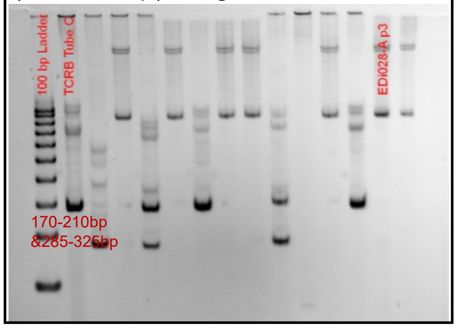

D) TCRG Tube A(145-255bp)
Target: Vg1-8, Vg10+Jg1.3/2.3

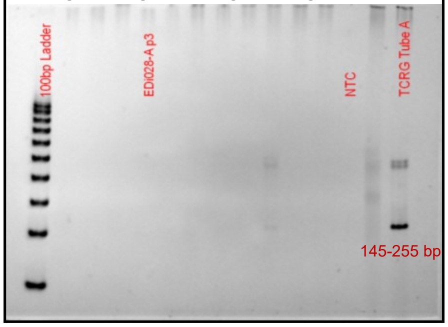

E) TCRG Tube B(80-220bp)
Target: Vg9 and Vg11+ Jg1.3/2.3

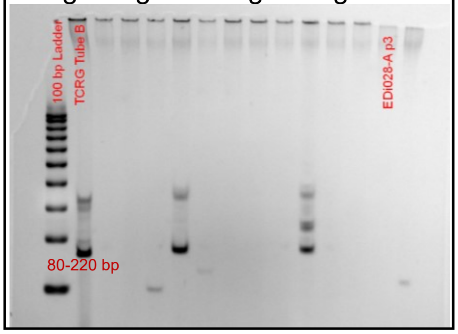

F) Specimen Control Size Ladder
Target: Multiple Genes

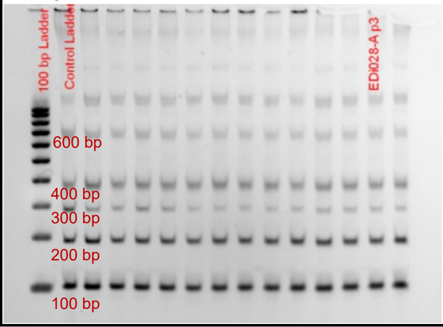

G)

Line	Passage	TCRB	TCRG
EDi021-A	p17	+	+
EDi022-A	p4	-	-
EDi023-A	p3	-	-
EDi025-A	p25	-	-
EDi026-A	p20	-	+
EDi027-A	p5	-	-
EDi028-A	p3	-	-
EDi029-A	p4	-	-
EDi030-A	p6	-	-
EDi031-A	p23	-	-
EDi032-A	p4	-	-
EDi033-A	p17	-	-
EDi034-A	p21	-	-
EDi035-A	p4	+	+
EDi036-A	p6	-	-
EDi037-A	p6	-	-
EDi038-A	p12	-	-
EDi039-A	p7	+	+
EDi040-A	p4	-	-
EDi041-A	p4	-	-
EDi042-A	p6	-	-
EDi043-A	p3	-	-
EDi044-A	p4	-	-
EDi045-A	p19	-	-

sFig26: Data showing T-cell clonal lineage for LBC iPSCs. For each line, three targets (Vß +Jß1/2, Vß +Jß2 and Dß +Jß1/2) were tested for T-Cell Receptor Beta Chain (TCRB), and two targets (Vg1-8, Vg10+Jg1.3/2.3; Vg9 and Vg11+ Jg1.3/2.3) for T-Cell Receptor Gamma Chain (TCRG). A-B-C) Representative 6% TBE Gel image for TCRB. D-E) Representative 6% TBE Gel image for TCRG. F) Representative 6% TBE Gel image for Control Genes. G) Table listing each of the LBC iPSC lines, indicating positivity (+) or negativity (-) for each of the T-cell receptors. Lines were considered positive if any band was present in any of the samples.

sFig.27: Mycoplasma Testing

A

A

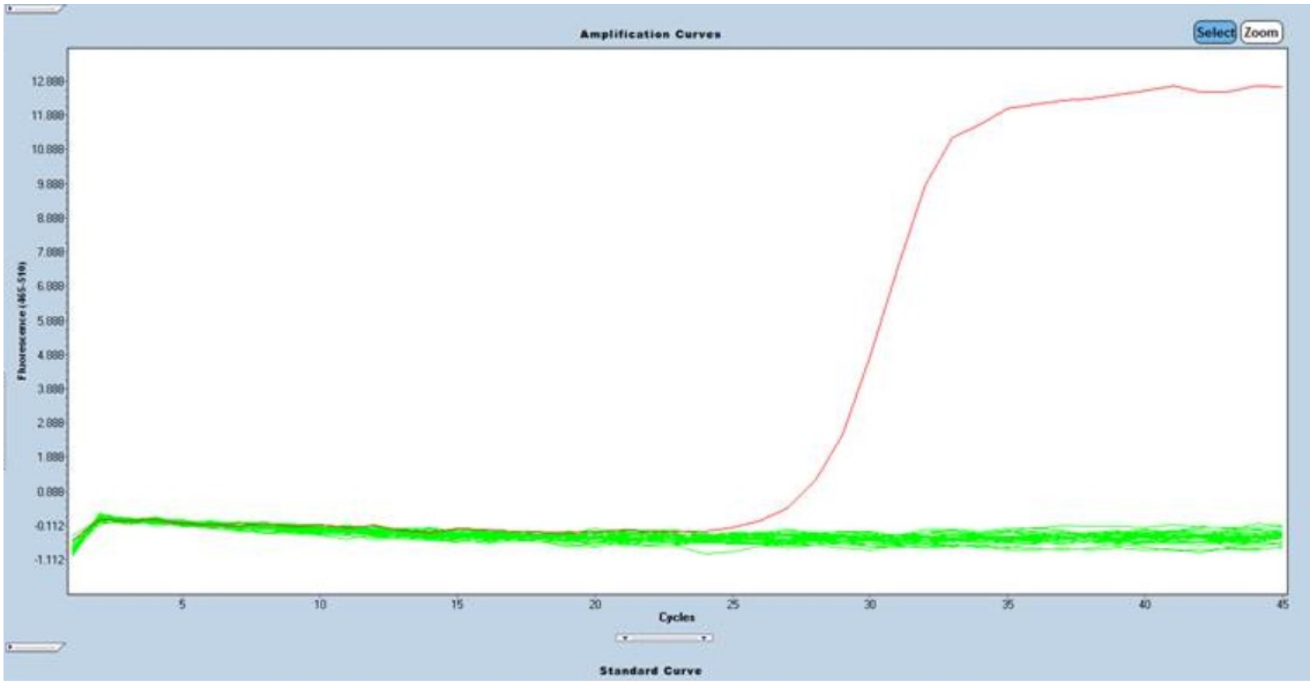

B

Sample	Passage	Luminescence Ratio	Mycoplasma
+ve control		48.78 (±25.06)	-
-ve control		0.07 (±0.04)	-
EDi021-A	p24	0.3	-
EDi022-A	p13	0.52	-
EDi023-A	p17	0.5	-
EDi025-A	p23	0.5	-
EDi026-A	p11	0.36	-
EDi027-A	p13	0.45	-
EDi028-A	p24	0.57	
EDi029-A	p14	0.38	-
EDi030-A	p14	0.4	-
EDi031-A	p29	0.45	-
EDi032-A	p11	0.45	-
EDi033-A			
EDi034-A	p17	0.46	-
EDi035-A	p17	0.47	-
EDi036-A	p13	0.54	-
EDi037-A	p20	0.39	-
EDi038-A			
EDi039-A			
EDi040-A	p17	0.47	-
EDi041-A	p38	0.19	-
EDi042-A	p17	0.43	-
EDi043-A			
EDi044-A	p13	0.41	-

sFig.27: Data showing the results of mycoplasma testing from iPSCs stored at two sites. **A)** PCR conducted by IDEXX BioAnalytics for samples provided by Cedars-Sinai Medical Center. All 24 LBC lines shown in green, positive control in red. **B)** Table showing results of MycoAlert Lonza LT07-318 assay conducted by Dementias Platform UK for available lines. Ratio values stated are the ratio of the luminescence signal of the kit substrate to that of the kit reagent. A ratio of 0-0.999 is negative for mycoplasma, 1–1.3 is borderline (requiring retest), and >1.3 is positive for mycoplasma. All samples tested at both sites were negative for mycoplasma.
